# Redesigning Aldolase Stereoselectivity by Homologous Grafting

**DOI:** 10.1371/journal.pone.0156525

**Published:** 2016-06-21

**Authors:** Carolin Bisterfeld, Thomas Classen, Irene Küberl, Birgit Henßen, Alexander Metz, Holger Gohlke, Jörg Pietruszka

**Affiliations:** 1 Institut für Bioorganische Chemie, Heinrich-Heine-Universität Düsseldorf im Forschungszentrum Jülich, 52426, Jülich, Germany; 2 Institute of Bio- and Geosciences IBG-1: Biotechnology, Forschungszentrum Jülich GmbH, 52425, Jülich, Germany; 3 Institute of Pharmaceutical and Medicinal Chemistry, Heinrich-Heine-Universität Düsseldorf, Düsseldorf, Germany; University of Cantebury, NEW ZEALAND

## Abstract

The 2-deoxy-d-ribose-5-phosphate aldolase (DERA) offers access to highly desirable building blocks for organic synthesis by catalyzing a stereoselective C-C bond formation between acetaldehyde and certain electrophilic aldehydes. DERA´s potential is particularly highlighted by the ability to catalyze sequential, highly enantioselective aldol reactions. However, its synthetic use is limited by the absence of an enantiocomplementary enzyme. Here, we introduce the concept of homologous grafting to identify stereoselectivity-determining amino acid positions in DERA. We identified such positions by structural analysis of the homologous aldolases 2-keto-3-deoxy-6-phosphogluconate aldolase (KDPG) and the enantiocomplementary enzyme 2-keto-3-deoxy-6-phosphogalactonate aldolase (KDPGal). Mutation of these positions led to a slightly inversed enantiopreference of both aldolases to the same extent. By transferring these sequence motifs onto DERA we achieved the intended change in enantioselectivity.

## Introduction

Protein engineering offers the opportunity to alter protein properties such as stability, substrate specificity, and conversion rates, respectively. However, addressing stereoselectivity still remains challenging for such endeavors, and this holds especially true in cases with no enantiocomplementary enzyme being available. In a summary of successful changes of enzyme enantioselectivity, Faber, Kazlauskas and coworkers described that these were achieved in most cases by altering the substrate binding mode [1]. Notably, even a single amino acid exchange can be sufficient to invert stereoselectivity as demonstrated, e.g., for thiamine diphosphate-dependent enzymes [2, 3]. The key step for rational protein design is the identification of these positions for mutagenesis. According to a study of Kazlauskas et al., amino acids close to the active site are often the more effective points of attack for changing enantioselectivity [4]. A fruitful approach to identify these stereoselectivity-determining positions is the analysis of enzyme families to expose conserved amino acids as summarized by Pleiss [5]. However, understanding the molecular mechanism of an enzyme´s enantiopreference is still challenging, even when a crystal structure is known. Grafting of specific patterns from related enzymes can be an alternative to generate a protein with the desired function [6, 7]. Referring to enzyme selectivity, Pazmiño et al. could identify amino acid positions, where variations led to an improved enantioselectivity by comparison of two sequence-related cyclopentanone monooxygenases, with one protein structure being modeled [8].

Enantiocomplementarity is essential for organic synthesis endeavors: enantiomerically pure building blocks are required in both enantiomeric forms. In particular, the aldol motif is a key to many synthetic endeavors. Besides conventional aldol reactions, the use of aldolases as biocatalysts became popular during the last two decades. Especially the 2-deoxy-d-ribose-5-phosphate aldolase (DERA) is of interest because it catalyzes aldol reactions between the nucleophilic acetaldehyde and certain electrophilic aldehydes. Interestingly, DERA catalyzes sequential aldol reactions facilitating access to lactols with up to two newly formed stereogenic centers in a single sequence [9–13]. However, the synthetic use of DERA is limited by the lack of a known natural enantiocomplementary DERA. The chemoselectivity of DERA was already enhanced by Jennewein et al., allowing DERA to accept chloroacetaldehyde as a substrate [14], and Wong and coworkers improved DERA´s use of non-phosphorylated electrophiles [15]. However, so far no alteration of DERA´s enantio- or diastereoselectivity has been attempted. Thus, the stereoinversion of DERA is optimal for applying and testing the grafting approach from homologous aldolases.

**Fig 1:**
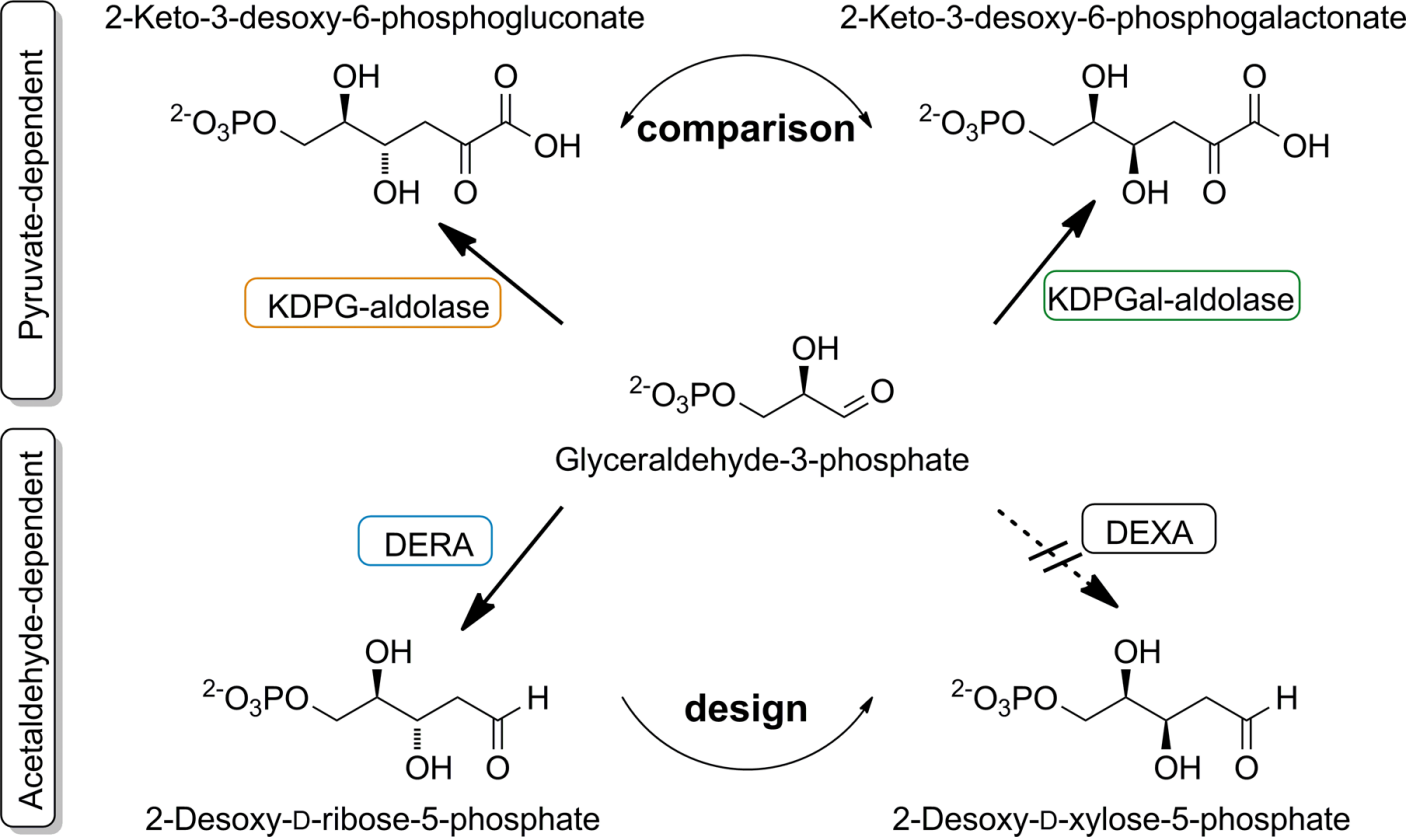
**The natural reaction of the enantiocomplementary pyruvate–dependent aldolases KDPG and KDPGal (top) and of DERA (bottom).** An enzyme that is diastereoselectively complementary to DERA (“DEXA” = 2-deoxy-d-xylose-5-phosphate aldolase) does not exist in nature as indicated by the dashed line.

We identified amino acid positions responsible for enantioselectivity and activity by comparing homologous aldolases, the enantiocomplementary pyruvate-dependent 2-keto-3-deoxy-6-phosphogluconate aldolase (KDPG) and 2-keto-3-deoxy-6-phosphogalactonate aldolase (KDPGal). All three aldolases are (αβ)_8_-barrel (TIM-barrel) proteins and, as class I aldolases, form an intermediate *Schiff* base between a particular lysine in their active site and their nucleophilic substrate during catalysis. While using a common electrophile, glyceraldehyde-3-phosphate, these aldolases merely differ by their specificity for a certain nucleophile, acetaldehyde for DERA and pyruvate for KDPG and KDPGal (Fig 1). In our homologous grafting approach we used geometric analyses and molecular dynamics (MD) simulations of KDPG and KDPGal to identify their determinants of stereoselectivity, which were then transferred onto DERA, leading to the intended change in its enantioselectivity.

## Materials and Methods

### Molecular Dynamics Simulations

The crystallographic protein structures of KDPG (PDB ID: 1eun [16]) and KDPGal (PDB ID: 2v81 [17]) were obtained from the Protein Data Bank PDB [18]. For KDPG, which forms a trimer in the crystallographic structure, only chain A was used. Crystallographic waters assigned to the prepared protein chains were retained for the simulation. Sulfate ions were removed. Selenomethionines were replaced by methionines, retaining the crystallographic conformation. Protein hydrogen atoms and side chain conformations of asparagines, glutamines, and histidines were assigned by REDUCE [19]. Hydrogens of crystal waters were added by the xLEaP program from the AMBER 9 package of molecular simulations programs [20].

MD simulations (MD) were performed with the AMBER 9 package of molecular simulation programs [20] using the Cornell et al. force field [21] with modifications introduced by Hornak et al. (ff99SB) [22]. Protein structures including crystallographic waters were solvated in a truncated octahedron of TIP3P water [23] such that the distance between the edges of the box and the closest solute atom was at least 11 Å. Periodic boundary conditions were applied using the particle mesh Ewald (PME) method [24] to treat long-range electrostatic interactions. Bond lengths of bonds to hydrogen atoms were constrained by SHAKE [25, 26]. The time step for all MD simulations was 2 fs, and a direct-space non-bonded cutoff of 8 Å was applied. After minimization the system was heated over 50 ps from 100 K to 300 K using canonical ensemble (NVT) MD. Then, the solvent density was adjusted over 50 ps using isothermal-isobaric ensemble (NPT) MD. Positional restraints applied during thermalization (with a force constant of 5 kcal mol^-1^ Å^-2^) were reduced in a stepwise manner over 50 ps followed by 50 ps of unrestrained canonical ensemble (NVT) MD at 300 K with a time constant of 2 ps for heat bath coupling to a Berendsen thermostat. Production runs were carried out for 10 ns; snapshots of the structure were saved every 1 ps. Interatomic distances were calculated for all saved snapshots by the PTRAJ program from the AMBER 9 package of molecular simulations programs [20].

### Biological Procedures

DNA manipulation: Enzymes, PCR reagents, and oligonucleotides were purchased from standard suppliers. PCR reactions were performed in 100 μl thin-wall tubes using a *VWR* Doppio gradient-thermocycler. DNA amounts were determined using a *Thermo Fischer Scientific* NanoDrop 2000c spectrophotometer. Commercially available kits were applied for DNA isolation and purification. DNA sequencing was conducted at SEQLAB or GATC Biotech. All primers are listed in S1 Table.

#### Cloning of KDPG_EC_ aldolase

The gene eda_EC_ encoding for KDPG from *E*. *coli* was amplified from the isolated genomic DNA of the strain DH5α using the primer pair edaEC_NdeI_fw and edaEC_XhoI_rv together with *Pfu* polymerase (10 min 95°C; 35x[45 s 95°C; 45 s 62°C; 45 s 72°C]; 10 min 72°C). The amplified DNA products were restricted by restriction endonucleases *NdeI* and *XhoI* and ligated into the likewise restricted and dephosphorylated vector pET22b (*Novagen*). The aldolase gene was expressed cytosolically with a C-terminally fused hexa-histidine tag. The obtained vector is referred to as pET22b::eda.

#### Generation of KDPG_EC_-variants

According to the method of Tillett and coworkers a sequence and ligation independent cloning was carried out to obtain the mutants [27]. Two pairwise PCRs were carried out using *Phusion* polymerase (*ThermoScientific Fischer*) and the primer pairs edaEC_chimI_fw and edaEC_chimI_rv_t as well as edaEC_chimI_fw_t and edaEC_chimI_rv, respectively (30 s 98°C; 25x[10 s 98°C; 30 s 55–65°C; 2 min 72°C]; 10 min 72°C; template: pET22b::eda). After the PCR the two mixes were combined and used for transformation of chemically competent *E*. *coli* DH5α. To introduce mutation T161V the same procedure was used with edaEC_T161V_fw and edaEC_T161V_rv_t as well as edaEC_T161V_fw_t and edaEC_T161V_rv, respectively, on the templates pET22b::eda or pET22b::eda_chimI (see above) to obtain both the single and the double mutant.

#### Cloning of KDPGal_EC_ aldolase

The procedure for the gene encoding for KDPGal_EC_ was exactly the same as for the gene encoding for KDPG_EC_ but with the primer pair dgoaEC_NdeI_fw and dgoaEC_XhoI_rv. The obtained vector is referred to as pET22b::dgoa.

#### Generation of KDPGal_EC_-variants

A *round-the-horn* PCR was used to implement the mutations onto the KDPGal gene [28–30]. The inverse PCR was carried out using *Phusion* polymerase onto pET22b::dgoa as template and as oligonucleotides dgoaEC_chimI_fw and dgoaEC_chimI_rv [6 min 95°C; 35x(30 s 95°C; 30 s 45–65°C; 1.5 min 70°C); 10 min 70°C]. The vector pET22b::dgoa_chimI was obtained. The same procedure was used with dgoaEC_V154T_fw and dgoaEC_V154T_rv onto pET22b::dgoa and pET22b::dgoa_chimI to obtain both the single and the double mutant.

#### Generation of a DERA_EC_ library

The gene deoC_EC_ encoding *E*. *coli* DERA_EC_ was amplified (10 min 95°C; 35x[45 s 95°C; 45 s 61°C; 45 s 72°C]; 10 min 72°C) from *E*. *coli* K12 chromosomal DNA using deocEC_NdeI_fwd and deocEC_EcoRI_rev as primers (S1 Table #59 and #60). The ligation with pET-21a(+) was done using the sticky ends generated by *NdeI* and *EcoRI*. The resulting construct is pET-21a::deoc_EC_ wt [31]. A DERA enzyme library consisting of 49 variants was constructed by exchanging the amino acid codons for T18, L20, and A203 within the wildtype (wt) gene to codons for 12 amino acids (R, N, D, C, G, H, I, L, F, S, Y, V, except for L20N and L20C) and deleting the amino acid codons for G204 and G205 as well as pairwise combinations of these single mutations (S1 Fig).

General Protocol for QuikChange PCR: PCR reactions were performed in a total volume of 50 μl containing 10 μl 5x Phusion HF buffer, 2 U Phusion^TM^ polymerase, ca. 30 ng template DNA, 200 μM dNTPs, 0.2 μM of each primer, and water. The applied temperature protocol was 30 sec at 95°C, 16 cycles of 30 sec at 95°C, 1 min at 50–65°C, and 6 min at 68°C, with a final step of 10 min at 68°C. Amplification was confirmed by agarose gel electrophoresis (1% agarose gels). PCR products were incubated with 10 U DpnI at 37°C overnight to digest template DNA.

General Protocol for Round-The-Horn PCR (RTH PCR): PCR reactions were performed in a total volume of 20 μl containing 4 μl 5x Phusion HF buffer, 0.4 U Phusion^TM^ polymerase, ca. 30 ng template DNA, 200 μM dNTPs, 6.25 nM of each primer, and water. The applied temperature protocol was 30 sec at 98°C, 25 cycles of 10.2 sec at 98°C, 30 sec at 55–70°C, and 2 min at 72°C, with a final step of 10 min at 70°C. Amplification was confirmed by agarose gel electrophoresis (1% agarose gels). Ligation with 2 μl T4 ligation buffer and 1 μl T4 ligase was performed at room temperature for 1 h. The ligated PCR products were incubated with 10 U DpnI at 37°C overnight to digest template DNA.

General Procedure for Plasmid Preparation: Chemically competent *E*. *coli* DH5α cells were transformed with the PCR products, followed by selection on LB agar plates supplemented with Ampicillin. Inoculation of a 5 ml overnight culture with LB medium supplemented with Ampicillin by a single colony enabled plasmid preparation.

#### Expression and purification of the pyruvate-dependent aldolases

For recombinant, homologous expression of KDPG or KDPGal and their variants, chemically competent cells [32] of *E*. *coli* BL21(DE3) were transformed with the respective vectors and selected on LB agar plates supplemented with ampicillin. In a baffled 3 l *Fernbach* flask 1 l TB-medium [1.2% (*w/v*) Trypton, 2.4% (*w/v*) yeast extract, 0.4% (*v/v*) glycerol, 0.231% (*w/v*) KH_2_PO_4_, and 1.254% (*w/v*) K_2_HPO_4_, 100 μg ml^-1^ ampicillin] were inoculated with 1% (*v/v*) of an overnight culture. The culture was incubated for 9 h at 20°C (120 rpm), induced with 0.1 mM *iso*-propylthio galactoside (IPTG), and incubated likewise for additional 15 h. Cells were harvested by centrifugation and stored at -20°C.

Frozen cells (up to 12.5 g) were thawed by suspending in purification buffer [20% cells (*w*/*v*)] and disrupted using a FRENCH press Cell Disruptor Cell *(Thermo Electron*, *Oberhausen GmbH)*. The crude extract was centrifuged for 20 min at 20,000 rcf and 4°C to obtain the cell free crude extract, which was applied to a 5 ml Ni-NTA column equilibrated with purification buffer (*Qiagen*, *Hilden*). The chromatographic elution was carried out using an *ÄKTA Purifier System* (*GE Healthcare*, *Munich*): 6 CV purification buffer with 0 mM imidazole; 6 CV purification buffer with 30 mM imidazole, and elution with 6 CV purification buffer with 250 mM imidazole. The eluate was concentrated at 4°C using *Vivaspin 20 MWCO 10 kDa* ultra-concentrators (*Sartorius Stedim Biotech S*.*A*., *France*). Imidazole was finally removed by *PD-10* desalting columns (*GE Healthcare*, *Munich*) equilibrated on purification buffer. Up to 150 mg purified protein was obtained from 1 l TB-medium. Purification buffer: 20 mM potassium phosphate, pH 6.5. Elution buffer concentrate: 20 mM potassium phosphate, 2 M imidazole/HCl, pH 6.5. Purification samples of KDPG and KDPGal aldolase were analyzed by subjecting equal amounts of protein (determined by Bradford assay [33]) to the SDS-PAGE using colloidal Coomassie stain [34] (S2 Fig).

#### Expression and purification of DERA wt and variants

Recombinant expression of DERA was performed in 3 l baffled *Fernbach* flasks using *E*. *coli* BL21(DE3) cells. Chemically competent cells were transformed with the respective DERA-vectors, followed by selection on LB plates supplemented with ampicillin. 1 l terrific broth (TB) with 100 μg ml^-1^ ampicillin was inoculated with a 5 ml overnight culture prepared from a single colony and grown at 37°C. After incubation at 25°C and 120 rpm for 8 h, expression of *deoC* gene was induced by the addition of 0.1 mM IPTG. After prolonged incubation of 15 h at 25°C, cells were harvested. For disruption using Ultrasonic Desintegrator Sonoplus HP 2070 (*Bandelin*, *Berlin*), the cells were suspended 20% (*w/v*) in triethanol amine buffer (100 mM, pH 7). Centrifugation at 18,000 rcf and 4°C resulted in cell free crude extract. For purification 2 ml of the cell free crude extract were loaded on a Q Sepharose HP column (5 ml) applied in an *ÄKTA Purifier System* (*GE Healthcare*, *Munich*) and eluted with triethanol amine buffer (100 mM, pH 7). The eluate was concentrated at 4°C using *Vivaspin 20 MWCO 10 kDa* ultra-concentrators (*Sartorius Stedim Biotech S*.*A*., *France*).

### Stereoselectivity Screening for Pyruvic Acid-Dependent Aldolases

For the stereoselectivity screening assay, the protein was isolated as described in the “biological procedures”. 50 mg of purified protein were dissolved in 10 ml of 20 mM potassium phosphate, 1 M sodium pyruvate, pH 6.5. The reaction was started by addition of 200 μl propanal (**1**). This mixture was shaken for 16 min (300 rpm) in a 50 ml reaction tube at 30°C. To facilitate the lactonization of the aldol product **3**, 100 μl of concentrated sulfuric acid was added and the reaction mixture was heated (with closed lid) to 60°C for 30 min. After chilling to ambient temperature, 5 ml ethyl acetate was added for extraction. The extract was employed for chromatographic analysis. Samples without enzyme were used as negative controls. 2-Oxo-4-ethyl-γ-butyro-lactone (**4**): Geometrical configurations were assumed to be as present in the natural substrate, although CIP priority changes. GC: R_t_ (*R*-enantiomer) = 24.4 min; R_t_ (*S*-enantiomer) = 26.5 min; 0.6 bar H_2_; [60°C isothermic for 5 min, 5°C min^-1^ to 150°C (5 min isothermic)]; MS (EI, 70 eV): m/z (%) = 128 (21) [M^+^], 99 (66) [(M-Et)^+^], 83 (100), 72 (60), 57 (36); ^1^H-NMR (600 MHz, CDCl_3_): keto-form δ [ppm] = 1 (t, ^3^J_2’,1’_ = 7.42 Hz, 3 H, 2’-H); 1.79 (m, 1 H, 1’a-H); 1.84 (m, 1 H. 1’b-H); 2.51 (dd, ^2^J_3a,3b_ = 19.9 Hz, ^3^J_3a,4_ = 5.4 Hz, 1 H, 3a-H); 2.99 (dd, ^2^J_3b,3a_ = 19.9 Hz, ^3^J_3b,4_ = 7.3 Hz, 1 H, 3b-H); 4.8 (m, 1 H, 4-H); ^1^H-NMR (600 MHz, CDCl_3_): enol-form δ [ppm] = 0.93 (t, ^3^J_2’,1’_ = 1.9 Hz, 3 H, 2’-H); 1.67 (m, 1 H, 1’a-H); 1.72 (m, 1 H, 1’b-H); 4.84 (ddd, ^3^J_4,3_ = 1.9 Hz, ^3^J_4,1’a_ = 5.67 Hz, ^2^J_4,1’b_ = 7.17 Hz, 1 H, 4-H); 5.9 (s(broad), 1 H, O*H*); 6.14 (d, ^3^J_3,4_ = 1.9 Hz, 1 H, 3-H)

### Protocol of DERA-Catalyzed Reactions for Library Screenings

The DERA catalyzed aldol reaction of acetaldehyde and propanal to (*R*)- or (*S*)-hydroxypentanal was performed in analytical scale, followed by a derivatization with 2,4-dinitrophenylhydrazine (2,4-DNPH). The products were extracted and measured by chiral HPLC. A stock solution, containing 7 μl of propanal (0.10 mmol), 7 μl of acetaldehyde (0.12 mmol, 1.3 eq.), and 14 μl dimethyl sulfoxide (0.2 mmol, 2.0 eq.), were pipetted to 0.5 ml of triethanolamine buffer (0.1 M, pH 7). After addition of 20 μl enzyme solution (cell free crude extract), the tubes were shaken for 16 h at 25°C and 200 rpm. For the derivatization step a solution of 40 mg 2,4-DNPH (0.2 mmol) and 7 μl conc. HCl (0.31 mmol) in 0.5 ml dimethyl sulfoxide was added to the DERA reaction mixture and stirred for 2 h at 50°C, 300 rpm. Extraction and sample preparation for HPLC was done according to Dick et al. [35] Samples without enzyme as well as a reaction with *E*. *coli* cells transformed with an empty vector were used as negative controls.

HPLC measurements were performed on a Dionex instrument consisting of a LPG-3400A pump, a WPS-3000TSL autosampler, a DAD-3000 UV-detector, and a TCC-3000SD oven. The instrument was fitted with a Daicel Chiralpak IA (250 mm x 4.6 mm) adjusted to 25°C. As solvent, a 80:20 (v/v) *n*-heptane/2-propanol-mixture was used applying a flow rate of 0.5 ml min^-1^. Compounds were solved in 90:10 (*v*/*v*) *n*-heptane/2-propanol-mixtures and measured at 345 nm. HPLC conditions: *Chiralpak IA*, 0.5 ml*min^-1^, 80:20 (*v*/*v*) *n*-heptane/2-propanol, 345 nm: (*ent-R*)-**7** *t*_R_ = 70.9 min, (*ent-S*)-**7** *t*_R_ = 79.0 min. The reference substances were synthesized according to ref. [35].

### Kinetic measurements with DERA

The activity was measured for the retro-aldol reaction of the natural substrate 2-deoxy-d-ribose-5-phosphate to glyceraldehyde-3-phosphate, which is converted by auxiliary enzymes to glycerol-3-phosphate under NADH consumption. The reaction is monitored on a photometer at 340 nm. The assay was done according to Dick et al. [36].

## Results and Discussion

### Identification of Key-Residues for KDPG and KDPGal

**Fig 2:**
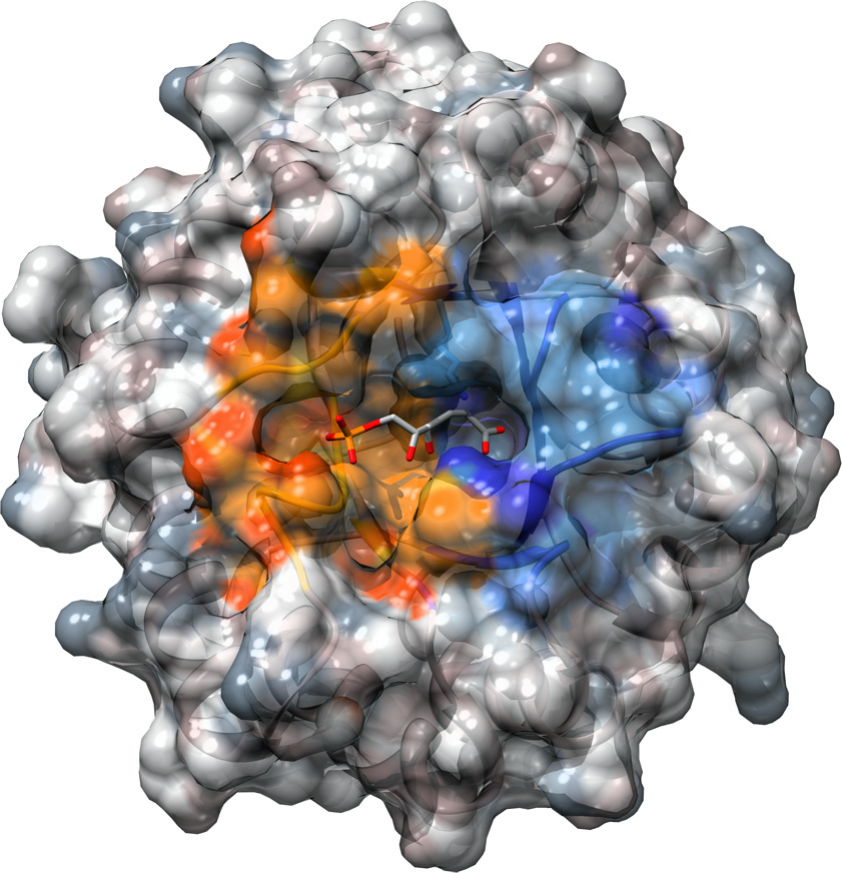
Top view on KDPGal (PDB ID: 2V82) [17]. The substrate is highlighted in stick representation. We hypothesize that the blue part of the protein is adapted to fix the nucleophilic carbonyl (here pyruvate), whereas the orange part is adapted to the electrophilic carbonyl (here glyceraldehyde-3-phosphate) and, thus, provides the basis for stereoselectivity.

DERA is the only aldolase using acetaldehyde as natural nucleophile (donor). Thus, there is no enantiocomplementary enzyme available. We therefore set out to learn from the homologous aldolases KDPG and KDPGal from *E*. *coli* how these enzymes accomplish enantioselectivity and use this knowledge to invert the enantioselectivity of DERA. Like DERA, these enzymes have an (αβ)_8_-architecture and belong to the type I aldolases. In contrast to DERA, KDPG and KDPGal aldolase utilize pyruvate rather than acetaldehyde as nucleophile. However, they naturally catalyze the hydrolysis of the respective 2-keto-3-deoxy-hexose acid to pyruvate and glyceraldehyde-3-phosphate, which is the same electrophile as in the natural DERA reaction (Fig 1). Our hypothesis is that the active sites of DERA as well as of KDPG and KDPGal consist of one part that is adapted to the nucleophile and another part that is adapted to the electrophile (Fig 2). The molecular basis of stereoselectivity should be inherent to the electrophilic part of the active site, which is presumably similar in both classes of aldolases, because the nucleophile adopts the very same position independent of the kind of pyruvate-dependent aldolase. Thus, the first step in designing an enantiocomplementary DERA was to identify stereodeterminants in the electrophilic part of the active site of the pyruvate-dependent aldolases. The identified stereodeterminants shall then be transferred onto DERA to alter its stereoselectivity.

**Fig 3:**
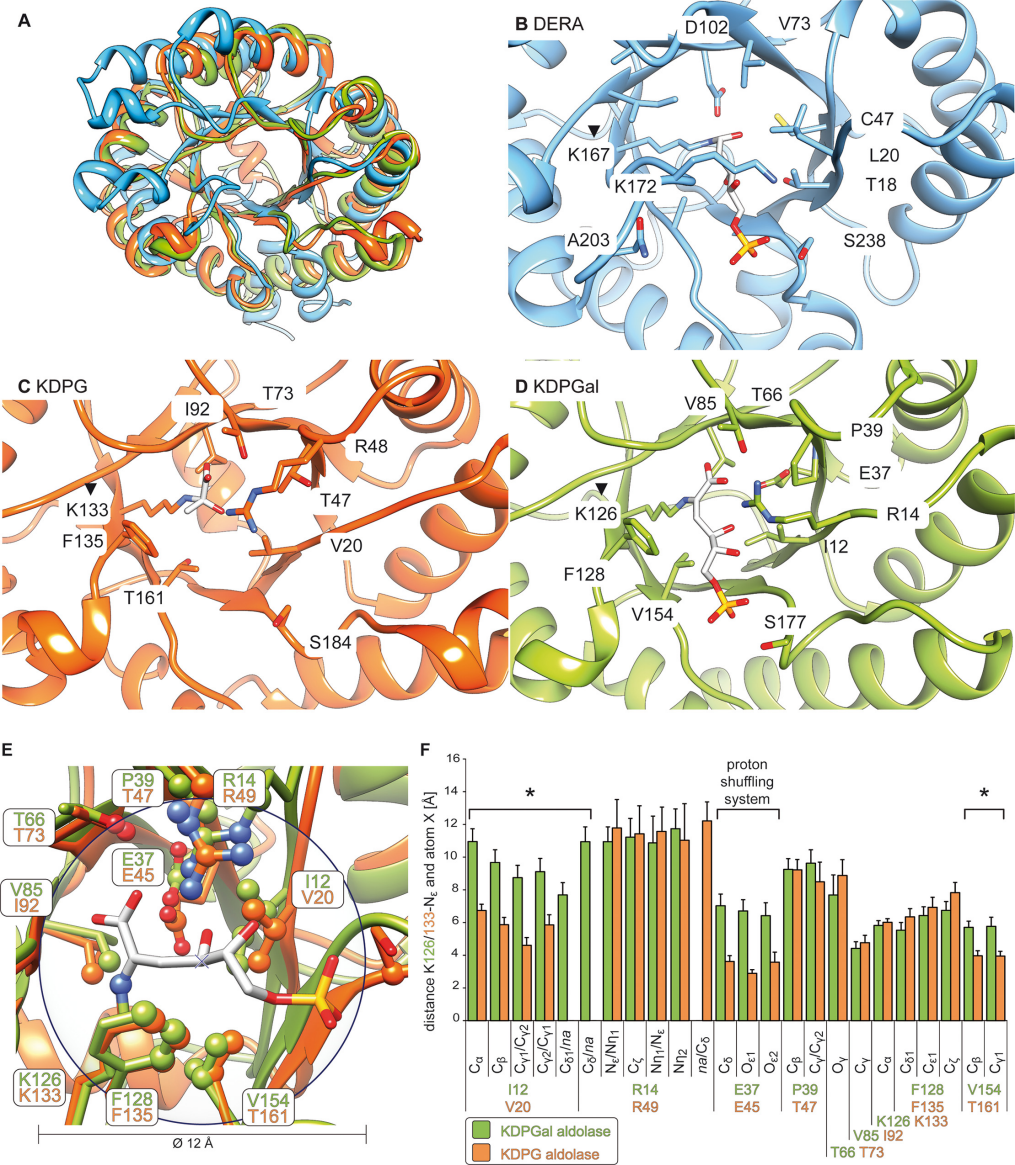
Geometric analyses of homologous aldolases. (A) – Superposition of DERA (PDB ID: 1JCL, blue) [37], KDPG (PDB ID: 1EUA, orange) [16], and KDPGal (PDB ID:2V82, green) [17] illustrates the structural similarity of these TIM-barrel proteins. (B-D) – Close-up views of the active sites of the three proteins. The active lysine is marked by a triangle (▼). (E) – Superposition of the active sites of KDPG and KDPGal aldolase. Atoms within a radius of 6 Å around the newly formed stereogenic center are indicated as spheres. (F) – Distances from MD simulations between the ε-amino group of the active lysine and all atoms within the 12 Å-Ø-sphere around the newly formed stereogenic center for both the KDPG and KDPGal aldolase, respectively. The mean values over all conformations of the MD simulation are given; bars indicate the standard deviation.

Slight conformational variations within the active site may affect the respective enantioselectivity. Hence, MD simulations were conducted to account for the dynamic behavior of the active sites. The MD simulations were carried out over 10 ns with snapshots extracted every 1 ps. The stereoselectivity-determining region was defined as all atoms within 6 Å radius of the newly formed stereogenic center (Fig 3E). The geometry of the active site was analyzed by calculating the distance of particular atoms to the ε-amino group of the active lysine as a reference point as to where the substrate would be present.

Analyses of the MD simulations revealed that most measured distances in the active sites of KDPG and KDPGal are similar. However, some amino acids at homologous sequence positions have significantly different distances to the ε-amino group of the active lysine, namely I12/V20 (in β_1_-sheet), E37/E45 (in β_2_-sheet), and V154/T161 (in β_7_-sheet), in the order KDPGal/KDPG aldolase respectively (Fig 3A–3D and 3F). The glutamate residues E37/E45 are part of the essential proton shuttling system enabling the formation of the nucleophilic enamine species. Hence, we assumed that major interactions are formed between these glutamates and the nucleophile, but not to the newly formed stereogenic center. Thus, we focused our design efforts on the two remaining sites, I12/V20 and V154/T161.

Site V154/T161 was previously shown to affect stereoselectivity in KDPG and KDPGal aldolase, respectively [17, 38–41]. To investigate this, we introduced V154T_KDPGal_ and T161V_KDPG_ single point mutations. So far, the I12/V20 site has not been investigated as to its influence on the stereoselectivity. However, crystal structures (Fig 3) suggest that single point mutations of this site may not be sufficient to affect stereoselectivity because the β_1_-sheet places V20_KDPG_ even closer to the active lysine than the more extended homologous I12_KDPGal_. In particular, P19_KDPG_ caused a kink in the β_1_-sheet, that way pushing V20_KDPG_ deeper into the active site pocket than I12_KDPGal_. Thus, to test the influence of the I12/V20 site, we transferred the entire β_1_-sheet of KDPG to KDPGal (chimβ_1;KDPGal_: V17L, V18I, P19A, V20I, I21L) and vice versa (chimβ_1;KDPG_: L9V, I10V, A11P, I12P, L13I). Finally, combinations of the single point mutations and the β-sheet transfer were introduced (chimβ_1_ T161V_KDPG_ and chimβ_1_ V154T_KDPGal_).

**Fig 4:**
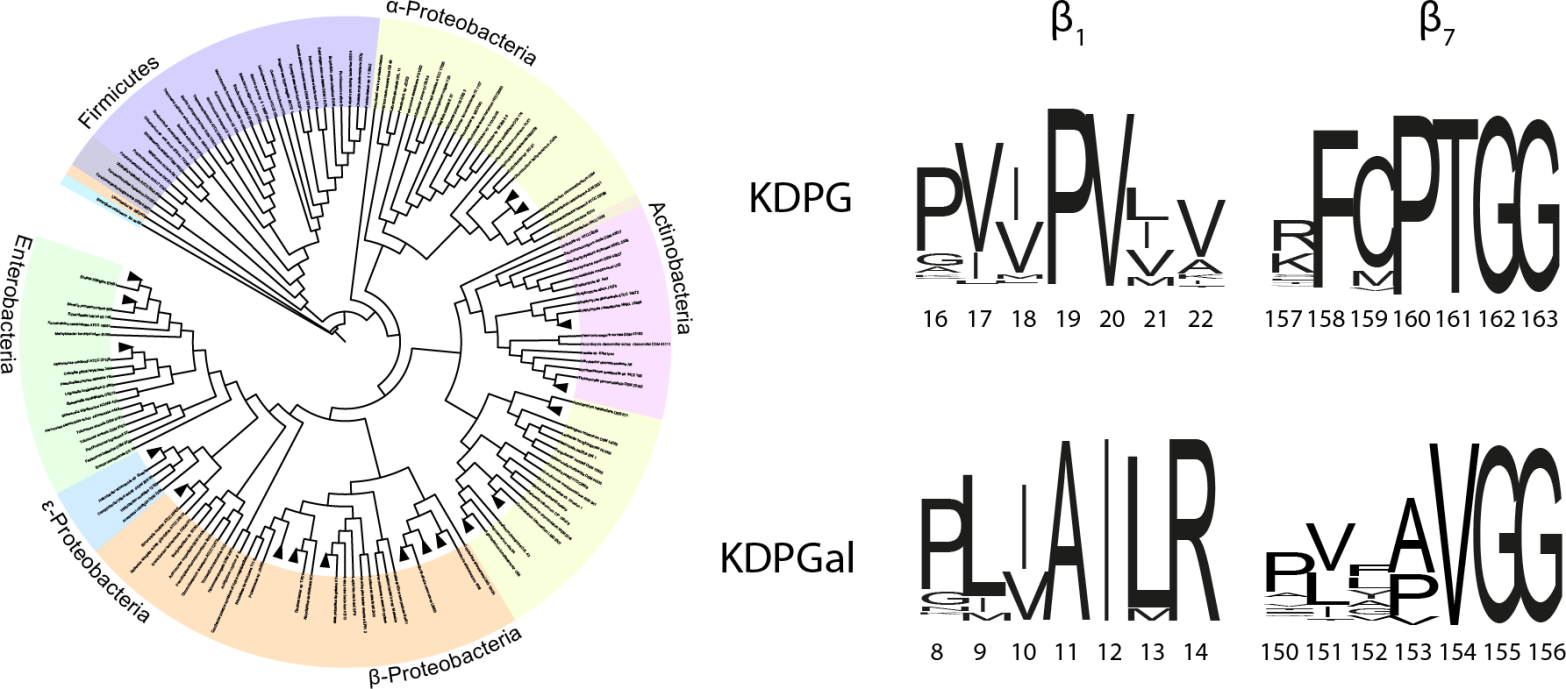
Phylogenetic analyses of the β-strands 1 and 7 from bacterial KDPG and KDPGal aldolases. The phylogenetic tree has been created using iTOL and shows the phylogenetic distribution KDPG/KDPGal-sequences used for identifying the respective patterns (~6000 sequences) [42]. The sequence logos have been created using the WebLogo server [43, 44]. The overall height of the stack indicates the sequence conservation at that position, while the height of individual symbols indicates the relative frequency of each amino acid at that position.

To further support the suggested mutation sites, we conducted phylogenetic analyses of bacterial KDPG and KDPGal aldolases (Fig 4). The I12/V20 site found by MD simulations is virtually fully conserved throughout all sequences, whilst the degree of conservation in the surrounding patches varies strongly. The P19 in KDPG mentioned above is highly conserved as is the homologous position A11 KDPGal. This supports our hypothesis that single amino acid exchanges at the I12/V20 site alone are insufficient to affect the stereoselectivity.

### Altering Stereoselectivity in Pyruvate-Dependent Aldolases

**Fig 5:**
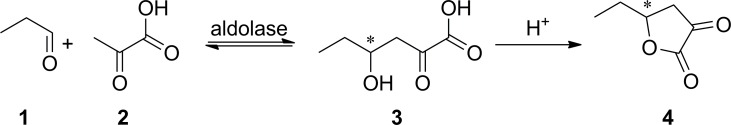
Model aldol reaction for testing the stereoselectivity of KDPG, KDPGal, and specific mutants. The aldol reaction of propanal (**1**) and pyruvic acid (**2**) leads to the enantioselective generation of **3** and, by acidic lactonization, to butyrolactone **4**.

The effect of the introduced mutations on the aldolases´ stereoselectivity was investigated by enzymatic reactions in synthetic (aldol) direction using pyruvic acid (**2**) as nucleophile and propanal (**1**) as electrophile (Fig 5). This approach has various advantages over measuring the aldolases´ enzymatic activity using their natural substrates (2-keto-3-deoxy-gluconate/galactonate-6-phosphate). First, conducting the assay in aldol direction yields an effective enantiomeric excess (*ee*) under competitive conditions and not an apparent value from comparative steady-state kinetics. Second, using the simplified substrate propanal leads to a simplified product portfolio, whereas the natural electrophile glyceraldehyde-3-phosphate would yield mixtures of lactones, furanoses, and pyranoses including the respective diastereomers.

Either the mutation in the β_7_-sheet or the transferred strand β_1_ affected the stereoselectivity (decrease of *ee* by up to -49%*ee*; Table 1). Combining both modifications strongly enhanced the effect and resulted in a slightly reversed stereoselectivity of KDPG (*ee* = -5%) and KDPGal (*ee* = 10%). Comparing these results with those from Walters et al., the enantioselectivities [log(E)] could be reproduced with the same tendency for the known mutants. However, for the uncompetitive assay used by Walters et al. the selectivities were much more optimistic [45, 46].

**Table 1:** Results of the stereoselectivity assay with the variants of the pyruvate-dependent aldolases KDPG and KDPGal. Own experiments have been carried out as triplicates from different enzyme-lots; standard-deviations are given for each *ee*. A negative control without enzyme did not show any product formation. Complex mutations: chimβ_1;KDPGal_: V17L, V18I, P19A, V20I, I21L and chimβ_1;KDPG_: L9V, I10V, A11P, I12P, L13I.

KDPG	%*ee* (4)	log(E)*	log(E)^§^	KDPGal	%*ee* (4)	log(E)*	log(E)^§^
wt	91±2	1.33	4.6	wt	-96±3	-1.69	-2.3
T161V	56±3	0.55	-0.15	V154T	-79±4	-0.93	-0.85
chimβ_1_	79±1	0.93	-	chimβ_1_	-49±3	-0.47	-
chimβ_1_ T161V	-5±4	-0.04	-	chimβ_1_ V154T	10±2	0.09	-

* Calculated from the enantiomeric excess (*ee*) value for the formation of compound **7** (Fig 5) using the equation log(E) = log[(-1-*ee*)/(*ee*-1)]

^§^ Values from Walters et al. [17], deduced from steady-state kinetics with natural substrates in retro-aldol direction.

### Development of an Aldol-Screening for DERA

**Fig 6:**
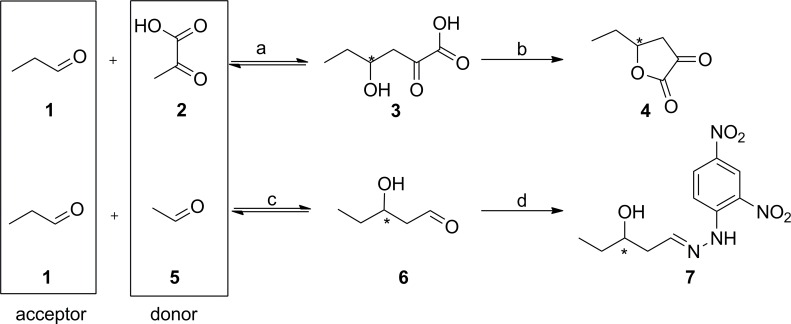
**Principle of KDPG, KDPGal, and DERA-catalyzed reactions for library screening** (a) KDPG and KDPGal aldolase, 275 mM propanal, 20 mM KP_i_, 1 M sodium pyruvate, pH 6.5, 30°C, 200 rpm, 18 h. (b) Acidified with sulfuric acid to pH 1. (c) 0.1 mmol propanal, 1.3 eq. acetaldehyde, 20 μl DERA crude extract, 0.5 ml triethanol amine buffer (0.1 M, pH7), 5% (v/v) dimethyl sulfoxide, 25°C, 200 rpm, 18 h. (d) 0.5 ml dimethyl sulfoxide, 2 eq. 2,4-dinitrophenylhydrazine, 3 eq. conc. HCl, 50°C, 2 h, 300 rpm.

One challenge was to develop a comparable screening method for KDPG/KDPGal aldolases and DERA. A screening in aldol direction should be applied to measure the *ee* of the newly formed stereogenic center (in comparison to a retro-aldol based assay [47, 48]). In order to design a comparable assay we used the same electrophile propanal (**1**) as above (Fig 5). The choice of the nucleophile is restricted by the corresponding enzymes, pyruvate for KDPG/KDPGal and acetaldehyde for DERA (Fig 6). The stereoselectivity of the respective DERA-catalyzed aldol reaction was assessed in a model reaction by the generation of both enantiomers of 3-hydroxypentanal (**6**). For KDPG and KDPGal aldolase, the products **4** were analyzed by gas chromatography using a chiral stationary phase. To be able to analyze the unstable aldol products **6** of DERA, they were derivatized. The formed hydrazones **7** [49–51] were extracted and measured by HPLC. Because this procedure may cause lot-specific differences during derivatization and extraction, each series of measurements included a triplicate measurement of DERA wt as a reference. The negative control without enzyme did not show any product formation.

A fixed volume of cell free crude extract of each DERA variant was used for the screening reactions whilst the expression level was controlled visually by means of SDS-PAGE (S3–S8 Figs). Only DERA variants T18D, T18C, and T18G had significantly lower expression levels than DERA wt, and for the L20H mutant virtually no soluble protein was expressed (see S1 Fig for details on the mutations).

### Transfer of Key-Residues onto Homologous DERA

As both sites I12/V20 (β_1_-sheet) and V154/T161 (β_7_-sheet) were confirmed as important determinants of KDPG stereoselectivity, we transferred them to corresponding sites in DERA. These corresponding sites were identified, based on a structural alignment, as T18 and L20 (both β_1_-sheet) as well as A203 (β_7_-sheet), respectively (Fig 3A). For a first generation of DERA variants, the respective sites were mutated using the degenerate codon NDT, that way including twelve different amino acids, in order to exhaustively explore the effect of these positions on stereoselectivity.

**Fig 7:**
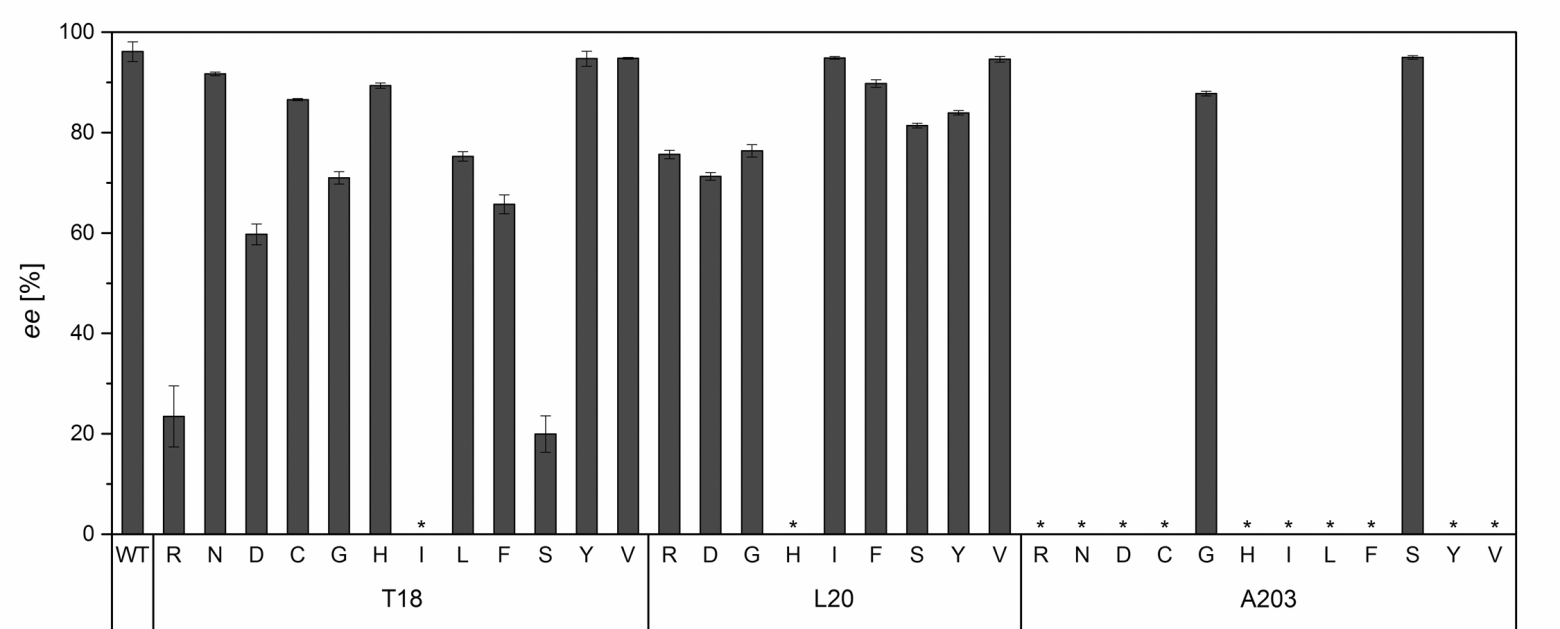
Enantiomeric excesses of the first generation variants of DERA at sites T18, L20, and A203, respectively. The *ee* was determined by the aldol screening (Fig 6) using chiral HPLC. The error bars represent the standard deviation of triplet measurements. A negative control without enzyme did not show any product formation. “*”: Results for variants with an activity lower than 2.0% of the DERA wt were omitted.

The results of the first generation of DERA variants show that T18 and L20 in the β_1_-sheet have a strong influence on DERA stereoselectivity (Fig 7). Especially T18S (*ee* = 20%) and T18R (*ee* = 23%) led to a decreased enantiomeric excess. These results support our hypothesis that the homologous sites are transferable and indicate that T18 is more important for stereoselectivity than L20.

Of the variants of the A203 position in the β_7_-sheet, only A203S and A203G showed sufficient activity to perform the aldol screening. The two variants contained amino acids of similar or smaller size than the original alanine. A203G showed the largest effect on stereoselectivity compared to DERA wt (*ee* = 88%); however, the overall effect is moderate compared to effects due to variants of the T18 position.

**Fig 8:**
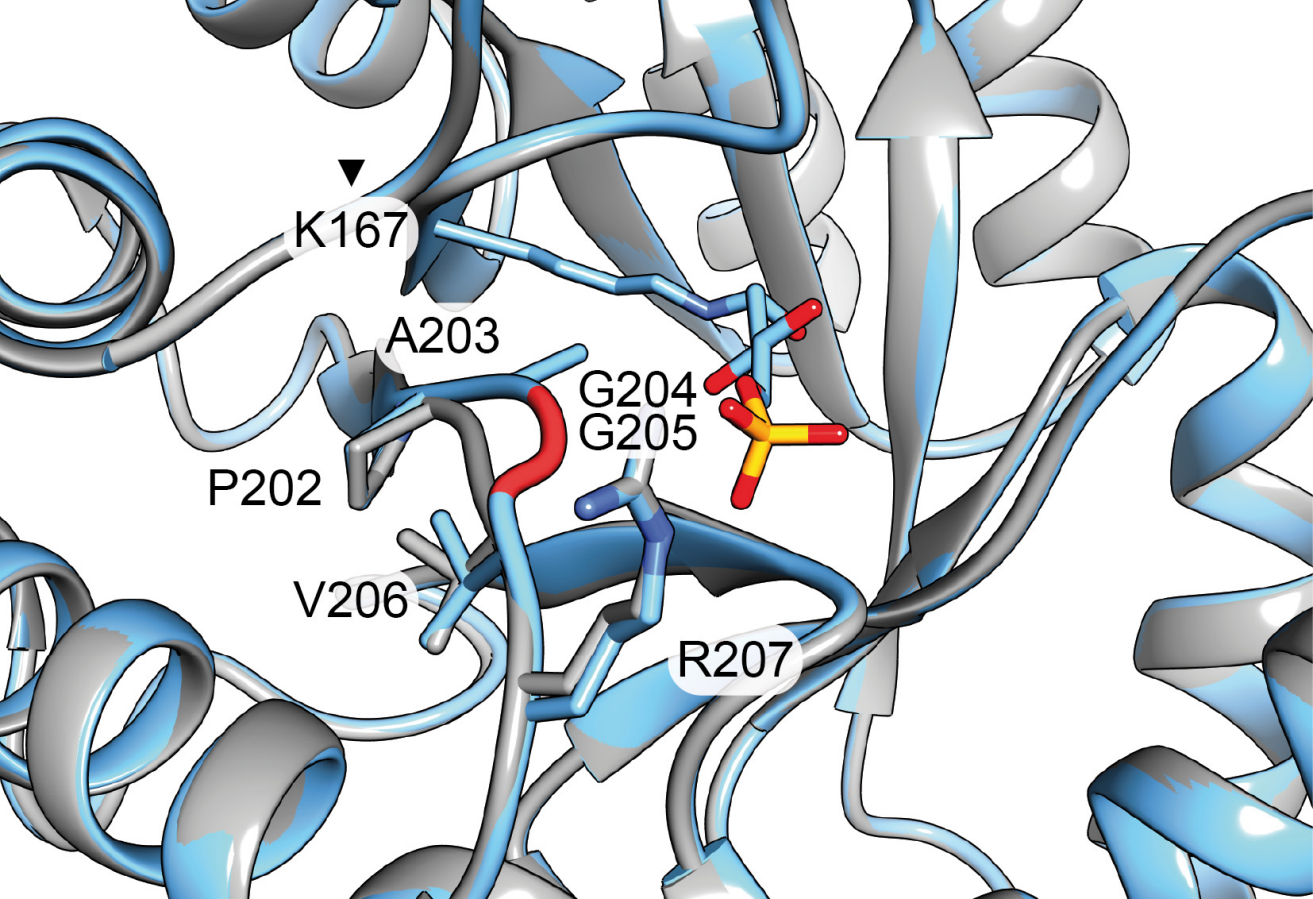
Comparison of DERA wt (blue) and the variant A203G ΔG204 ΔG205 (grey). The sites G204 and G205 are highlighted in red in the wt structure. The models were generated based on PDB ID 1JCL [37] using USCF Chimera [52].

With reference to these results, a second generation of DERA variants was constructed. Double mutations of the positions T18 and A203 were created. As there is no smaller amino acid than glycine, which resulted in the largest effect on stereoselectivity in A203G DERA, we aimed at further expanding the binding pocket for the electrophile by deleting the amino acids G204 and G205. Structural models generated from the DERA crystal structure by deleting these residues, changing A203 to glycine, and minimization indicated that these deletions (ΔG204, ΔG204 Δ205) should result in a retraction of the loop at the end of the β_7_-sheet (Fig 8). Furthermore, to study the effects of double mutations, combinations of the A203G and T18 variants were investigated.

**Fig 9:**
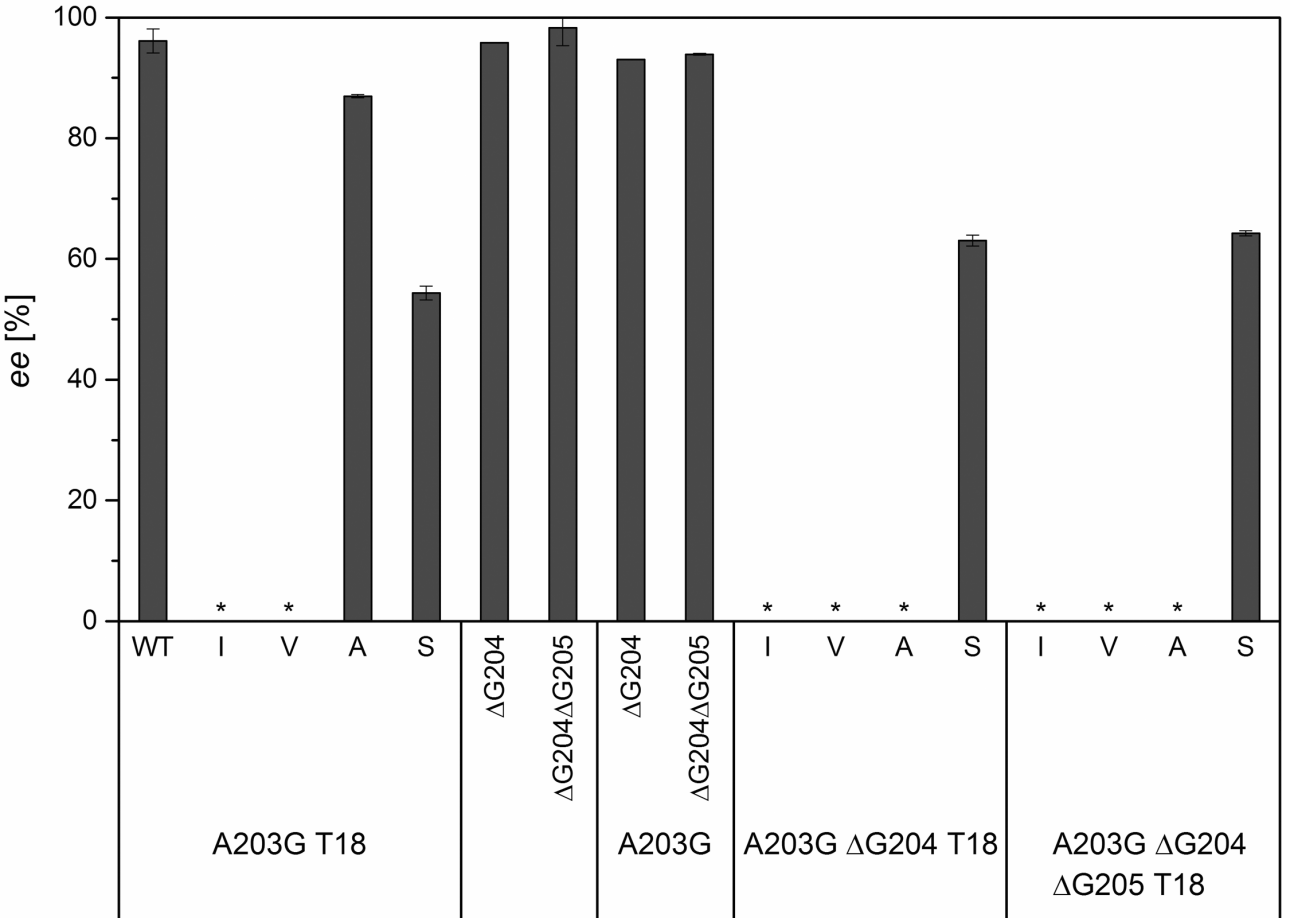
Enantiomeric excesses of the second generation of variants of DERA at sites T18 combined with A203G, A203G ΔG204, and A203G ΔG204 ΔG205, respectively. The *ee* was determined by the aldol screening (Fig 6) using chiral HPLC. The error bars represent the standard deviation of triple measurements. A negative control without enzyme did not show any product formation. “*”: Results for variants with an activity lower than 2.0% of the DERA wt were omitted.

The combination of A203G and T18S did not lead to a further decreased enantiomeric excess (*ee* = 54%). The deletion of G204 and G205 in combination with A203G, respectively, did not lead to a change in enantiomeric excess either. Finally, the combination of A203G, one or two glycine deletions, and mutation in position T18 to serine decreased the enantioselectivity significantly (*ee* = 63-64%, Fig 9), although this value is higher than that obtained for the A203G T18S variant above (ee = 54%).

Michaelis-Menten kinetics on the natural substrate of the DERA of the variant with the highest change in enantioselectivity T18S as well as the wildtype enzyme were performed. The results in Table 2 show an only ~3-fold decreased catalytic efficiency for T18S compared to the wildtype, leading to a catalytic efficiency of ~36 s^-1^ mM^-1^.

**Table 2:** Michaelis-Menten parameters from steady-state kinetics with 2-deoxy-d-ribose-5-phosphate (D5P) as substrate.

	K_M_ [mM]	k_cat_ [s^-1^]	k_cat_ K_M_^-1^[s^-1^ mM^-1^]
WT	0.29 ± 0.01	33.18 ± 0.66	114.72 ± 6.47
T18S	0.17 ± 0.01	6.05 ± 0.15	35.92 ± 2.59

## Conclusions

Enzymes are attractive catalysts because of their strict stereoselectivity. In some cases natural enantiocomplementary versions are available. Unfortunately, if an enantiocomplementary enzyme is missing, synthetic applications are severely restricted. Although DERA is such an example, this enzyme is still highly attractive for synthetic purposes [11, 53]. Various endeavors, such as rational enzyme design and directed evolution approaches, were employed for different enzymes to invert their stereoselectivity [54–60]. For the pyruvate dependent aldolases very convincing approaches for changing the stereoselectivity have been conducted [39, 40, 61]. However, to our knowledge, no alteration of the stereoselectivity of DERA has been described so far.

Here, we present an approach that aims at transferring the stereoselectivity-determining parts from homologous, pyruvate-dependent, and enantiocomplementary aldolases KDPG and KDPGal onto DERA. In order to apply this promising strategy, the stereoselectivity-determining sites of KDPG and KDPGal were identified using MD simulations, geometric, and phylogenetic analyses. For the pyruvate-dependent aldolases, we demonstrated the importance of I12/V20 (β_1_-sheet) and V154/T161 (β_7_-sheet) as stereoselectivity-determining sites, using a novel assay working in aldol instead of retro-aldol direction of the catalyzed reaction to elucidate the stereochemistry of the respective products. The advantage of the novel assay is that it is unbiased by non-competing effects contrary to previous assays exploiting the retro-aldol reaction in separate reactions of the two enantiomers [17]; as a result, for mutation sites previously described, lower stereoselectivity inversions compared to previous work were observed (Table 1). However, these obtained values reflect the synthetic capabilities more realistically. Although it was not possible to completely invert the enantioselectivity of KDPG or KDPGal, it was possible to pin-point I12/V20 (β_1_-sheet) and V154/T161 (β_7_-sheet) as essential residues for stereoselectivity. Variants of these restricted sites allowed not only to reduce stereoselectivity but even inverted it compared to KDPG (*ee* = -5%) and KDPGal (*ee* = 10%). This demonstrated that these sites are important for stereoselectivity.

We then transferred these sites onto DERA based on considerations derived from a structural superpositioning of the enzymes. The spatial positions of the stereoselectivity-determining sites should be the same because of their similar geometrical composition and their very same mode of action. Indeed, mutating the found sites caused a significant change in enantioselectivity within the first series of mutations (best *ee* found for T18S: 20%). Combinations of the identified stereoselectivity-determining sites did not further change the stereoselectivity. DERA has a very different mechanism in realizing its stereoselectivity compared to the homologous pyruvate-dependent aldolase, thus, additional stereoselectivity-determining sites might be involved as seen for the pyruvate-dependent aldolases. Hence, inversion of the stereoselectivity of DERA is likely an especially challenging problem; obviously further stereo-determinants of DERA must be characterized in future studies. However, the presented homologous grafting approach was shown to serve as a new entry point for difficult tasks for finding enantiocomplementary enzymes.

## Supporting Information

S1 Fig Overview of deoC_EC_ enzyme library construction. pET-21a(+)::deoC_EC_ T18X: The T18X variants were produced by RTH PCR using the deoC_EC_ wt construct as DNA-template and the different deoC_EC__T18X_fwd oligonucleotides as forward primers and deoC_EC__T18_rev as reverse primer (S1 Table #17–26). The T18X (X: I,S,V) variants were produced by QuikChange PCR using the deoC_EC_ wt construct as DNA-template and deoC_EC__T18I_fwd/deoC_EC_-T18S_fwd/deoC_EC_-T18V_fwd as forward primers and deoC_EC__T18I_rev/deoC_EC_-T18S_rev/deoC_EC_-T18V_rev as reverse primers (S1 Table #27–32). pET-21a(+)::deoC_EC_ L20X: The L20X variants were produced by RTH PCR using the deoC_EC_ wt construct as DNA-template and the different deoC_EC__L20X_fwd oligonucleotides as forward and deoC_EC__L20_rev as reverse primer (S1 Table #35–44). pET-21a(+)::deoC_EC_ A203X: The A203X variants were produced by RTH PCR using the deoC_EC_ wt construct as DNA-template and the deoC_EC__A203_fwd as forward and the degenerated oligonucleotide deoC_EC__L20NDT_rev as reverse primer (S1 Table #45 and #46). The A203X (X: R,D,C,S) variants were produced by RTH PCR using the deoC_EC_ wt construct as DNA-template and the deoC_EC__A203_fwd as forward and the different deoC_EC__A203X_rev oligonucleotides as reverse primer (S1 Table #45 and #47–50). pET-21a(+)::deoC_EC_ T18X,A203G: The variants with changes of amino acid position T18 and A203 were produced by QuikChange PCR using the pET-21a(+)::deoC_EC_ A203G plasmid as DNA-template and the different deoC_EC__T18X_fwd oligonucleotides as forward and the different deoC_EC_-T18X_rev oligonucleotides as reverse primers (S1 Table #27–34). The enzyme variants with either one or two glycine deletions were produced by RTH PCR using the deoC_EC_ wt construct as DNA-template and the oligonucleotides deoC_EC__ΔG204_fwd or deoC_EC__ΔG204,ΔG205_fwd as forward and the oligonucleotide deoC_EC__A203_rev as reverse primer (S1 Table #51–53). The variants with the A203G mutations and either one or two glycine deletions were produced by RTH PCR using the deoC_EC_ wt construct as DNA-template and the oligonucleotides deoC_EC__ΔG204_fwd or deoC_EC__ΔG204,ΔG205_fwd as forward and the oligonucleotide deoC_EC__A203G_rev as reverse primer (S1 Table #51–52 and #54). The enzyme variants with A203G, either one or two glycin deletions, and T18X (X: S,A,I,V) were produced by QuikChange PCR using the pET-21a(+)::deoC_EC_ A203G,ΔG204 or pET-21a(+)::deoC_EC_ A203G,ΔG204,ΔG205 plasmid as DNA-template and the different deoC_EC__T18X_fwd oligonucleotides as forward and the different deoC_EC_-T18X_rev oligonucleotides as reverse primers (S1 Table #27–34).
(TIF)

S2 Fig SDS-PAGE of purification samples of KDPG and KDPGal aldolase. Gel (NuPAGE 4–12% Bis-TRIS Gel, Invitrogen) with colloidal Coomassie stain. M – marker; CE – crude extract; CFCE – cell free crude extract; FT – chromatographic flow through; W_30_ – washing with 30 mM imidazole; E_250_ – elution with 250 mM imidazole. Equal amounts of protein (determined by Bradford assay) were subjected to the SDS-PAGE.
(TIF)

S3 Fig SDS-PAGE of DERA T18NDT variants. 4–12% Bis-TRIS Gel with colloidal Coomassie stain. 10 μL of a 1:100 dilution of cell-free crude extracts were used, the standard was Roti®-Mark 10–150. WT: DERA wildtype, the mutation is represented in the single-letter code.
(TIF)

S4 Fig SDS-PAGE of DERA L20NDT variants. 4–12% Bis-TRIS Gel with colloidal Coomassie stain. 10 μL of a 1:100 dilution of cell-free crude extracts were used, the standard was Roti®-Mark 10–150. WT: DERA wildtype, the mutation is represented in the single-letter code.
(TIF)

S5 Fig SDS-PAGE of DERA A203NDT variants. 4–12% Bis-TRIS Gel with colloidal Coomassie stain. 10 μL of a 1:100 dilution of cell-free crude extracts were used, the standard was Roti®-Mark 10–150. WT: DERA wildtype, the mutation is represented in the single-letter code.
(TIF)

S6 Fig SDS-PAGE of DERA A203G/T18X variants. 4–12% Bis-TRIS Gel with colloidal Coomassie stain. 10 μL of a 1:100 dilution of cell-free crude extracts were used, the standard was Roti®-Mark 10–150. WT: DERA wildtype, 29: DERA A203G/T18S, 30: DERA A203G/T18A, 31: DERA A203G/T18I, 32: DERA A203G/T18V.
(TIF)

S7 Fig SDS-PAGE of DERA variants with glycine deletions. 4–12% Bis-TRIS Gel with colloidal Coomassie stain. 10 μL of a 1:100 dilution of cell-free crude extracts were used, the standard was Roti®-Mark 10–150. WT: DERA wildtype, 1: DERA ΔG204, 2: ΔG204/ΔG205, 3: A203G/ΔG204, 4: A203G/ΔG204/ΔG205.
(TIF)

S8 Fig SDS-PAGE of DERA triple and quadruples variants. 4–12% Bis-TRIS Gel with colloidal Coomassie stain. 10 μL of a 1:100 dilution of cell-free crude extracts were used, the standard was Roti®-Mark 10–150. 1: DERA wildtype, 2: A203G/ΔG204/T18I, 3: A203G/ΔG204/T18V, 4: A203G/ΔG204/T18A, 5: A203G/ΔG204/T18S, 6: A203G/ΔG204/ΔG205/T18I, 7: A203G/ΔG204/ΔG205/T18V, 8: A203G/ΔG204/ΔG205/T18A, 9: A203G/ΔG204/ΔG205/T18S.
(TIF)

S1 Table Primers used for creation of KDPG, KDPGal, and DERA enzyme variants. [Phos] indicates a phosphorylation at the 5’ position.
(PDF)

## References

[1] Mugford PF, Wagner UG, Jiang Y, Faber K, Kazlauskas RJ. Enantiocomplementary Enzymes: Classification, Molecular Basis for Their Enantiopreference, and Prospects for Mirror‐Image Biotransformations. Angew Chem Int Ed. 2008; 47(46): 8782–93. 10.1002/anie.200705159 18850616

[2] Gocke D, Walter L, Gauchenova E, Kolter G, Knoll M, Berthold C, et al Rational Protein Design of ThDP-Dependent Enzymes—Engineering Stereoselectivity. Chem Express. 2008; 9(3): 406–12. 10.1002/cbic.200700598 18224647

[3] Hailes HC, Rother D, Müller M, Westphal R, Ward JM, Pleiss J, et al Engineering stereoselectivity of ThDP-dependent enzymes. Exp Op Therap Patents. 2013; 280(24): 6374–94. 10.1111/febs.12496 24034356

[4] Kazlauskas RJ, Bornscheuer UT. Finding better protein engineering strategies. Nat Chem Biol. 2009; 5(8): 526–9. 10.1038/nchembio0809-526 19620988

[5] Pleiss J. Systematic Analysis of Large Enzyme Families: Identification of Specificity- and Selectivity-Determining Hotspots. ChemBioChem. 2014; 6(4): 944–50.

[6] Donaldson JM, Zer C, Avery KN, Bzymek KP, Horne DA, Williams JC. Identification and grafting of a unique peptide-binding site in the Fab framework of monoclonal antibodies. P Natl Acad Sci USA. 2013; 110(43): 17456–61. 10.1073/pnas.1307309110 PMC3808661 24101516

[7] Reyda S, Sohn C, Klebe G, Rall K, Ullmann D, Jakubke H-D, et al Reconstructing the Binding Site of Factor Xa in Trypsin Reveals Ligand-induced Structural Plasticity. J Micro 9 2003; 325(5): 963–77. 10.1016/s0022-2836(02)01337-2 12527302

[8] Pazmiño DET, Snajdrova R, Rial DV, Mihovilovic MD, Fraaije MW. Altering the Substrate Specificity and Enantioselectivity of Phenylacetone Monooxygenase by Structure-Inspired Enzyme Redesign. Adv Synth Catal. 2007; 349(8–9): 1361–8.

[9] Liu J, Hsu C-C, Wong C-H. Sequential aldol condensation catalyzed by DERA mutant Ser238Asp and a formal total synthesis of atorvastatin. Tetrahedron Lett. 2004; 45(11): 2439–41.

[10] Gijsen HJM, Wong C-H. Unprecedented Asymmetric Aldol Reactions with Three Aldehyde Substrates Catalyzed by 2-Deoxyribose-5-phosphate Aldolase. J Am Chem Soc. 1994; 116(18): 8422–3.

[11] Wolberg M, Dassen BHN, Schürmann M, Jennewein S, Wubbolts MG, Schoemaker HE, et al Large-Scale Synthesis of New Pyranoid Building Blocks Based on Aldolase-Catalysed Carbon-Carbon Bond Formation. Adv Synth Catal. 2008; 350(11–12): 1751–9.

[12] Liu J, Wong C-H. Aldolase-Catalyzed Asymmetric Synthesis of Novel Pyranose Synthons as a New Entry to Heterocycles and Epothilones. Angew Chem. 2002; 114(8): 1462–5. 10.1002/1521-3773(20020415)41:8<1404::aid-anie1404>3.0.co;2-g 19750780

[13] Patel JM. Biocatalytic synthesis of atorvastatin intermediates. J Mol Catal B. 2009; 61(3–4): 123–8.

[14] Jennewein S, Schürmann M, Wolberg M, Hilker I, Luiten R, Wubbolts M, et al Directed evolution of an industrial biocatalyst: 2-deoxy-D-ribose 5-phosphate aldolase. Biotechnol J. 2006; 1(5): 537–48. 16892289 10.1002/biot.200600020

[15] DeSantis G, Liu J, Clark DP, Heine A, Wilson IA, Wong C-H. Structure-based mutagenesis approaches toward expanding the substrate specificity of D-2-deoxyribose-5-phosphate aldolase. Bioorg Med Chem. 2003; 11(1): 43–52. 12467706 10.1016/s0968-0896(02)00429-7

[16] Allard J, Grochulski P, Sygusch J. Covalent intermediate trapped in 2-keto-3-deoxy-6- phosphogluconate (KDPG) aldolase structure at 1.95-Å resolution. P Natl Acad Sci USA. 2001; 98(7): 3679–84. 10.1073/pnas.071380898 PMC31111 11274385

[17] Walters MJ, Srikannathasan V, McEwan AR, Naismith JH, Fierke CA, Toone EJ. Characterization and crystal structure of Escherichia coli KDPGal aldolase. Bioorg Med Chem. 2008; 16(2): 710–20. 17981470 10.1016/j.bmc.2007.10.043 PMC3326530

[18] Berman HM, Westbrook J, Feng Z, Gilliland G, Bhat TN, Weissig H, et al The Protein Data Bank. Nucleic Acids Res. 2000; 28(1): 235–42. 10592235 10.1093/nar/28.1.235 PMC102472

[19] Word JM, Lovell SC, Richardson JS, Richardson DC. Asparagine and glutamine: Using hydrogen atom contacts in the choice of side-chain amide orientation. J Mol Biol. 1999; 285(4): 1735–47. 9917408 10.1006/jmbi.1998.2401

[20] Case DA, Cheatham TE, Darden T, Gohlke H, Luo R, Merz KM, et al The Amber biomolecular simulation programs. J Comput Chem. 2005; 26(16): 1668–88. 16200636 10.1002/jcc.20290 PMC1989667

[21] Wang JM, Cieplak P, Kollman PA. How well does a restrained electrostatic potential (RESP) model perform in calculating conformational energies of organic and biological molecules? J Comput Chem. 2000; 21(12): 1049–74.

[22] Hornak V, Abel R, Okur A, Strockbine B, Roitberg A, Simmerling C. Comparison of multiple amber force fields and development of improved protein backbone parameters. Proteins: Struct, Funct, Bioinf. 2006; 65(3): 712–25. 10.1002/prot.21123 PMC4805110 16981200

[23] Jorgensen WL, Chandrasekhar J, Madura JD, Impey RW, Klein ML. Comparison of Simple Potential Functions for Simulating Liquid Water. J Chem Phys. 1983; 79(2): 926–35.

[24] Darden T, York D, Pedersen L. Particle Mesh Ewald—an N.Log(N) Method for Ewald Sums in Large Systems. J Chem Phys. 1993; 98(12): 10089–92.

[25] Ryckaert JP, Ciccotti G, Berendsen HJC. Numerical-Integration of Cartesian Equations of Motion of a System with Constraints—Molecular-Dynamics of N-Alkanes. J Comput Phys. 1977; 23(3): 327–41.

[26] Miyamoto S, Kollman PA. Settle—an Analytical Version of the Shake and Rattle Algorithm for Rigid Water Models. J Comput Chem. 1992; 13(8): 952–62.

[27] Chiu J, March PE, Lee R, Tillett D. Site-directed, Ligase-Independent Mutagenesis (SLIM): a single-tube methodology approaching 100% efficiency in 4 h. Nucl Acids Res. 2004; 32(21): e174 15585660 10.1093/nar/gnh172 PMC535700

[28] Douglas CC, Thomas D, Lanman J, Prevelige PE Jr. Investigation of N-terminal domain charged residues on the assembly and stability of HIV-1 CA. Biochemistry. 2004; 43(32): 10435–41. 15301542 10.1021/bi049359g

[29] Follo C, Isidoro C. A fast and simple method for simultaneous mixed site-specific mutagenesis of a wide coding sequence. Biotechnol Appl Biochem. 2008; 49(2): 175–83. 17640176 10.1042/BA20070045

[30] Moore SD, Prevelige PE Jr. A P22 scaffold protein mutation increases the robustness of head assembly in the presence of excess portal protein. J Virol. 2002; 76(20): 10245 12239300 10.1128/JVI.76.20.10245-10255.2002 PMC136566

[31] Kullartz I, Pietruszka J. Cloning and characterisation of a new 2-deoxy-d-ribose-5-phosphate aldolase from Rhodococcus erythropolis. J Biol Chem. 2012; 161(2): 174–80. 10.1016/j.jbiotec.2011.12.018 22222309

[32] Mülhardt C. Der Experimentator: Molekularbiologie/Genomics: Springer; 2008.

[33] Bradford MM. A rapid and sensitive method for the quantitation of microgram quantities of protein utilizing the principle of protein-dye binding. Anal Biochem. 1976; 72(1): 248–54. 942051 10.1016/0003-2697(76)90527-3

[34] Dyballa N, Metzger S. Fast and sensitive colloidal coomassie G-250 staining for proteins in polyacrylamide gels. Journal of visualized Experiments. 2009; (30). 10.3791/1431 PMC3149902 19684561

[35] Dick M, Weiergräber OH, Classen T, Bisterfeld C, Bramski J, Gohlke H, et al Trading off stability against activity in extremophilic aldolases. Scientific Reports. 2016; 6: 17908 10.1038/srep17908 26783049 PMC4725968

[36] Dick M, Hartmann R, Weiergraber OH, Bisterfeld C, Classen T, Schwarten M, et al Mechanism-based inhibition of an aldolase at high concentrations of its natural substrate acetaldehyde: structural insights and protective strategies. Chemical Science. 2016. 10.1039/c5sc04574f PMC6016325 30155096

[37] Heine A, DeSantis G, Luz JG, Mitchell M, Wong C-H, Wilson IA. Observation of Covalent Intermediates in an Enzyme Mechanism at Atomic Resolution. Sci Synth. 2001; 294(5541): 369–74. 10.1126/science.1063601 11598300

[38] Fong S, Machajewski TD, Mak CC, Wong C-H. Directed evolution of D-2-keto-3-deoxy-6-phosphogluconate aldolase to new variants for the efficient synthesis of D- and L-sugars. Chem Biol. 2000; 7(11): 873–83. 11094340 10.1016/s1074-5521(00)00035-1

[39] Baker P, Seah SYK. Rational Design of Stereoselectivity in the Class II Pyruvate Aldolase BphI. J Am Chem Soc. 2012; 134(1): 507–13. 10.1021/ja208754r 22081904

[40] Royer SF, Haslett L, Crennell SJ, Hough DW, Danson MJ, Bull SD. Structurally Informed Site-Directed Mutagenesis of a Stereochemically Promiscuous Aldolase To Afford Stereochemically Complementary Biocatalysts. J Am Chem Soc. 2010; 132(33): 11753–8. 10.1021/ja104412a 20684556

[41] Baerga-Ortiz A, Popovic B, Siskos AP, O'Hare HM, Spiteller D, Williams MG, et al Directed Mutagenesis Alters the Stereochemistry of Catalysis by Isolated Ketoreductase Domains from the Erythromycin Polyketide Synthase. Chem Biol. 2006; 13(3): 277–85. 16638533 10.1016/j.chembiol.2006.01.004

[42] Letunic I, Bork P. Interactive Tree Of Life v2: online annotation and display of phylogenetic trees made easy. Nucleic Acids Res. 2011; 39(suppl 2): W475–W8. 21470960 10.1093/nar/gkr201 PMC3125724

[43] Schneider TD, Stephens RM. Sequence logos: a new way to display consensus sequences. Nucleic Acids Res. 1990; 18(20): 6097–100. 2172928 10.1093/nar/18.20.6097 PMC332411

[44] Crooks GE, Hon G, Chandonia J-M, Brenner SE. WebLogo: a sequence logo generator. Genome Res. 2004; 14(6): 1188–90. 15173120 10.1101/gr.849004 PMC419797

[45] Walters MJ, Srikannathasan V, McEwan AR, Naismith JH, Fierke CA, Toone EJ. Characterization and crystal structure of *Escherichia coli* KDPGal aldolase. Bioorg Med Chem. 2008; 16(2): 710–20. 17981470 10.1016/j.bmc.2007.10.043 PMC3326530

[46] Cheriyan M, Toone EJ, Fierke CA. Improving upon Nature: Active Site Remodeling Produces Highly Efficient Aldolase Activity toward Hydrophobic Electrophilic Substrates. Biochemistry. 2012; 51(8): 1658–68. 10.1021/bi201899b 22316217 PMC3315183

[47] Bisterfeld C, Küberl I, Dick M, Pietruszka J. A Fluorogenic Screening for Enantio- and Diastereoselectivity of 2-Deoxy-d-ribose-5-phosphate Aldolases. Synlett. 2016; 27(01): 11–6.

[48] Greenberg WA, Varvak A, Hanson SR, Wong K, Huang H, Chen P, et al Development of an efficient, scalable, aldolase-catalyzed process for enantioselective synthesis of statin intermediates. P Natl Acad Sci USA. 2004; 101(16): 5788–93. 10.1073/pnas.0307563101 PMC395986 15069189

[49] Pilz J, Meineke I, Gleiter CH. Measurement of free and bound malondialdehyde in plasma by high-performance liquid chromatography as the 2,4-dinitrophenylhydrazine derivative. J Chromatogr B. 2000; 742(2): 315–25. 10.1016/s0378-4347(00)00174-2 10901136

[50] Zwiener C, Glauner T, Frimmel F. Method optimization for the determination of carbonyl compounds in disinfected water by DNPH derivatization and LC–ESI–MS–MS. Anal Bioanal Chem. 2002; 372(5–6): 615–21. 11941429 10.1007/s00216-002-1233-y

[51] van Leeuwen SM, Hendriksen L, Karst U. Determination of aldehydes and ketones using derivatization with 2,4-dinitrophenylhydrazine and liquid chromatography–atmospheric pressure photoionization-mass spectrometry. J Chromatogr. 2004; 1058(1–2): 107–12. 10.1016/j.chroma.2004.08.149 15595657

[52] Pettersen EF, Goddard TD, Huang CC, Couch GS, Greenblatt DM, Meng EC, et al UCSF Chimera—a visualization system for exploratory research and analysis. J Comput Chem. 2004; 25(13): 1605–12. 15264254 10.1002/jcc.20084

[53] Müller M. Chemoenzymatic Synthesis of Building Blocks for Statin Side Chains. Angew Chem Int Ed. 2005; 44(3): 362–5. 10.1002/anie.200460852 15593081

[54] Rüthlein E, Classen T, Dobnikar L, Schölzel M, Pietruszka J. Finding the Selectivity Switch–A Rational Approach towards Stereocomplementary Variants of the Ene Reductase YqjM. Adv Synth Catal. 2015; 357(8): 1775–86.

[55] Hall M, Stueckler C, Ehammer H, Pointner E, Oberdorfer G, Gruber K, et al Asymmetric Bioreduction of C = C Bonds using Enoate Reductases OPR1, OPR3 and YqjM: Enzyme-Based Stereocontrol. Adv Synth Catal. 2008; 350(3): 411–8.

[56] Sun Z, Lonsdale R, Kong X-D, Xu J-H, Zhou J, Reetz M. Reshaping an Enzyme Binding Pocket for Enhanced and Inverted Stereoselectivity: Use of Smallest Amino Acid Alphabets in Directed Evolution. Angew Chem Int Ed. 2015; 54(42): 12410–5. 10.1002/anie.201501809 25891639

[57] Lin H, Tang D-F, Ahmed AAQ, Liu Y, Wu Z-L. Mutations at the putative active cavity of styrene monooxygenase: Enhanced activity and reversed enantioselectivity. J Biol Chem. 2012; 161(3): 235–41. 10.1016/j.jbiotec.2012.06.028 22796094

[58] Wada M, Hsu C-C, Franke D, Mitchell M, Heine A, Wilson I, et al Directed evolution of N-acetylneuraminic acid aldolase to catalyze enantiomeric aldol reactions. Bioorg Med Chem. 2003; 11(9): 2091–8. 12670660 10.1016/s0968-0896(03)00052-x

[59] Chen-Goodspeed M, Sogorb MA, Wu F, Raushel FM. Enhancement, Relaxation, and Reversal of the Stereoselectivity for Phosphotriesterase by Rational Evolution of Active Site Residues†. Biochemistry. 2001; 40(5): 1332–9. 11170460 10.1021/bi001549d

[60] Smith M, Smith MEB, Hibbert E, Jones A, Dalby P, Hailes H. Enhancing and Reversing the Stereoselectivity ofEscherichia coliTransketolaseviaSingle-Point Mutations. Adv Synth Catal. 2008; 350(16): 2631–8.

[61] Williams GJ, Woodhall T, Farnsworth LM, Nelson A, Berry A. Creation of a Pair of Stereochemically Complementary Biocatalysts. J Am Chem Soc. 2006; 128(50): 16238–47. 17165777 10.1021/ja065233q

