# Supplementary figures and images for: Redesigning Aldolase Stereoselectivity by Homologous Grafting
Source: PLoS One. 2016 Jun 21;11(6):e0156525. doi: 10.1371/journal.pone.0156525 (PMC4915726; doi:10.1371/journal.pone.0156525)

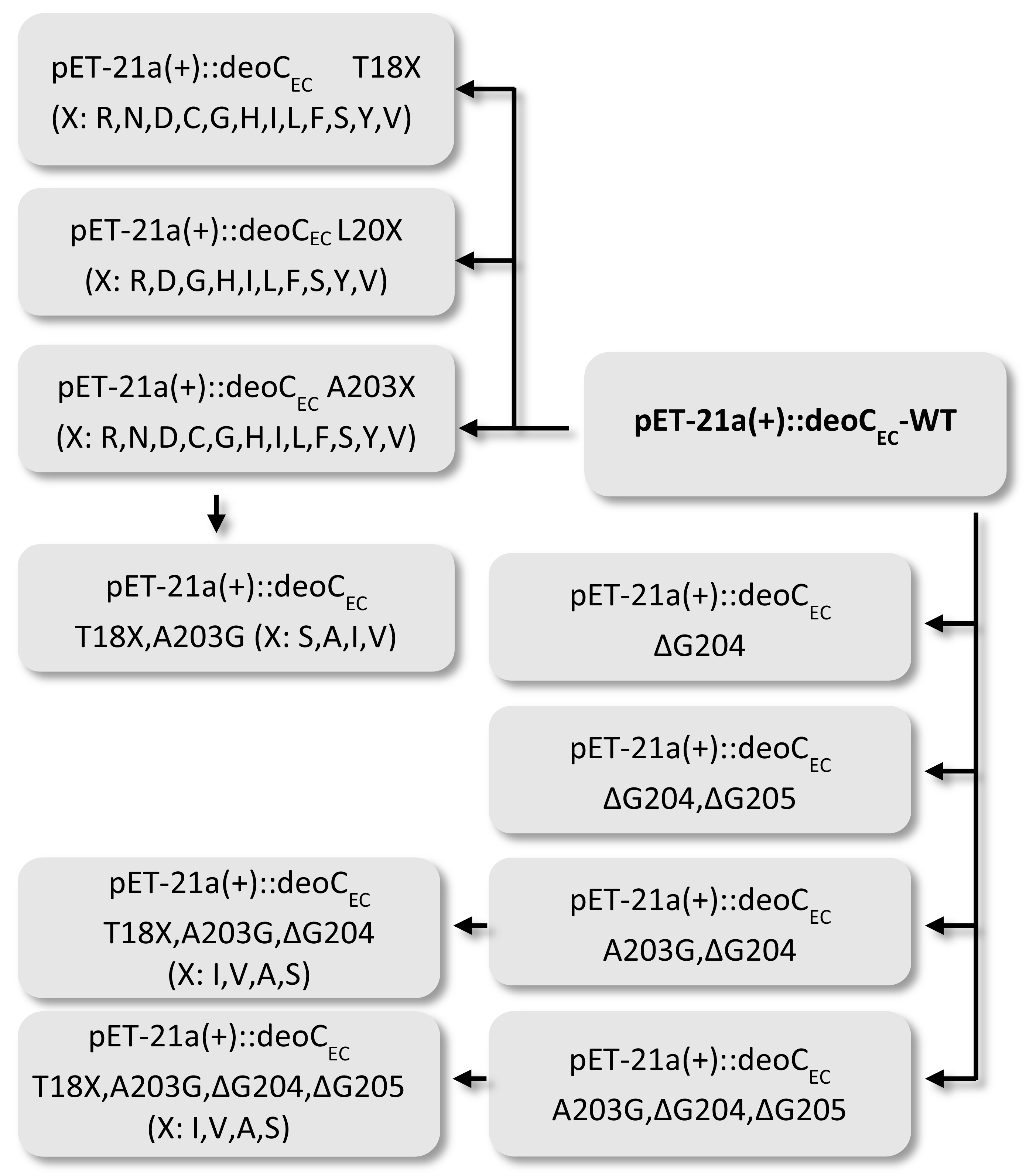

Supplement: S1 Fig — pET-21a(+)::deoCEC T18X: The T18X variants were produced by RTH PCR using the deoCEC wt construct as DNA-template and the different deoCEC_T18X_fwd oligonucleotides as forward primers and deoCEC_T18_rev as reverse primer (S1 Table #17–26). The T18X (X: I,S,V) variants were produced by QuikChange PCR using the deoCEC wt construct as DNA-template and deoCEC_T18I_fwd/deoCEC-T18S_fwd/deoCEC-T18V_fwd as forward primers and deoCEC_T18I_rev/deoCEC-T18S_rev/deoCEC-T18V_rev as reverse primers (S1 Table #27–32). pET-21a(+)::deoCEC L20X: The L20X variants were produced by RTH PCR using the deoCEC wt construct as DNA-template and the different deoCEC_L20X_fwd oligonucleotides as forward and deoCEC_L20_rev as reverse primer (S1 Table #35–44). pET-21a(+)::deoCEC A203X: The A203X variants were produced by RTH PCR using the deoCEC wt construct as DNA-template and the deoCEC_A203_fwd as forward and the degenerated oligonucleotide deoCEC_L20NDT_rev as reverse primer (S1 Table #45 and #46). The A203X (X: R,D,C,S) variants were produced by RTH PCR using the deoCEC wt construct as DNA-template and the deoCEC_A203_fwd as forward and the different deoCEC_A203X_rev oligonucleotides as reverse primer (S1 Table #45 and #47–50). pET-21a(+)::deoCEC T18X,A203G: The variants with changes of amino acid position T18 and A203 were produced by QuikChange PCR using the pET-21a(+)::deoCEC A203G plasmid as DNA-template and the different deoCEC_T18X_fwd oligonucleotides as forward and the different deoCEC-T18X_rev oligonucleotides as reverse primers (S1 Table #27–34). The enzyme variants with either one or two glycine deletions were produced by RTH PCR using the deoCEC wt construct as DNA-template and the oligonucleotides deoCEC_ΔG204_fwd or deoCEC_ΔG204,ΔG205_fwd as forward and the oligonucleotide deoCEC_A203_rev as reverse primer (S1 Table #51–53). The variants with the A203G mutations and either one or two glycine deletions were produced by RTH PCR using the deoCEC wt construct as DNA-template and th [file pone.0156525.s001.tif]

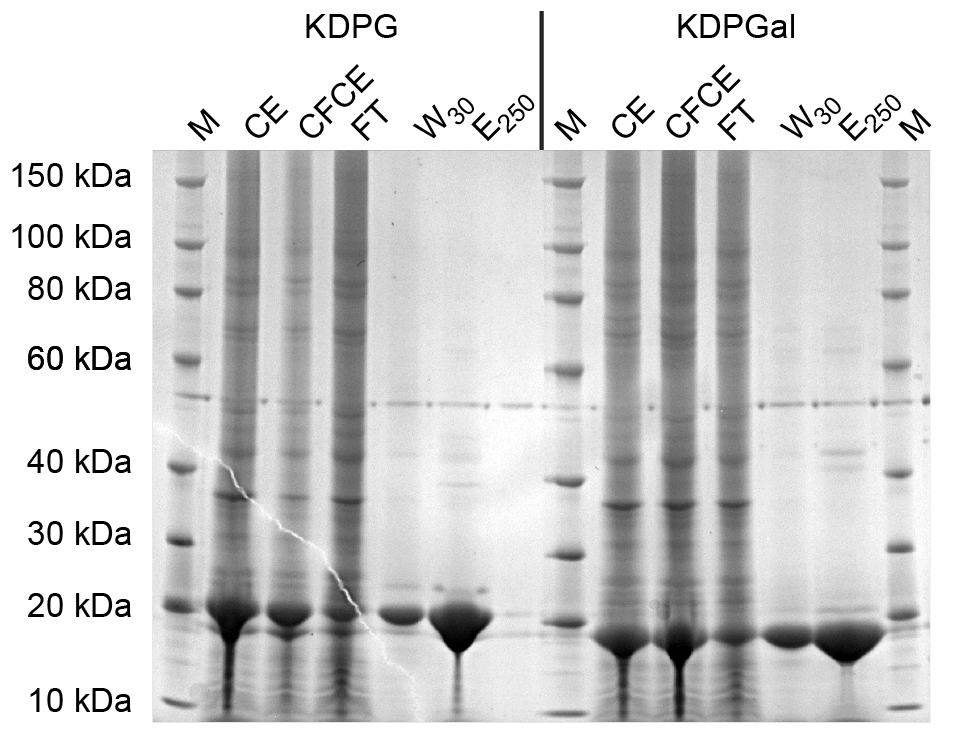

Supplement: S2 Fig — Gel (NuPAGE 4–12% Bis-TRIS Gel, Invitrogen) with colloidal Coomassie stain. M – marker; CE – crude extract; CFCE – cell free crude extract; FT – chromatographic flow through; W30 – washing with 30 mM imidazole; E250 – elution with 250 mM imidazole. Equal amounts of protein (determined by Bradford assay) were subjected to the SDS-PAGE. (TIF) [file pone.0156525.s002.tif]

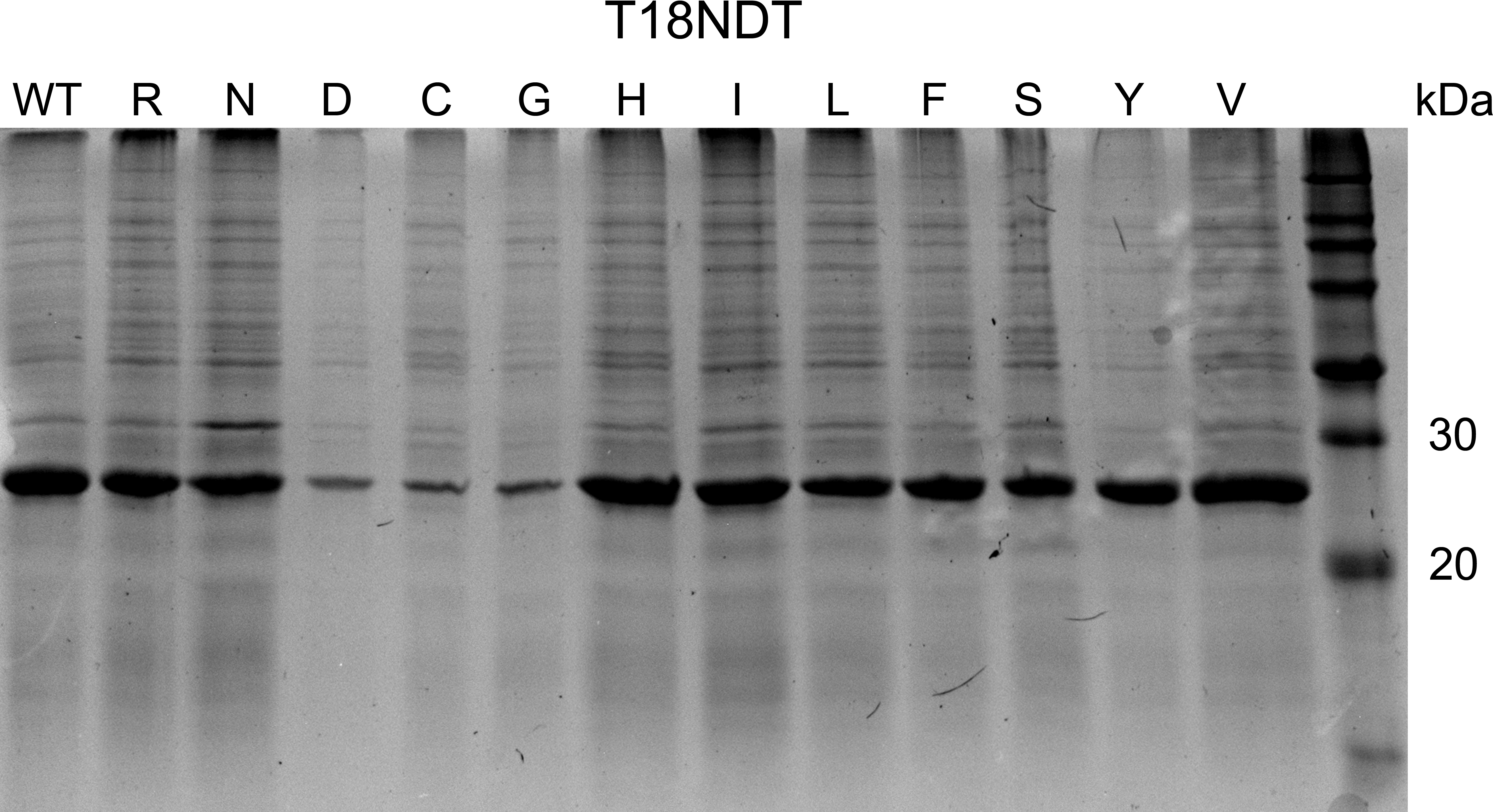

Supplement: S3 Fig — 4–12% Bis-TRIS Gel with colloidal Coomassie stain. 10 μL of a 1:100 dilution of cell-free crude extracts were used, the standard was Roti®-Mark 10–150. WT: DERA wildtype, the mutation is represented in the single-letter code. (TIF) [file pone.0156525.s003.tif]

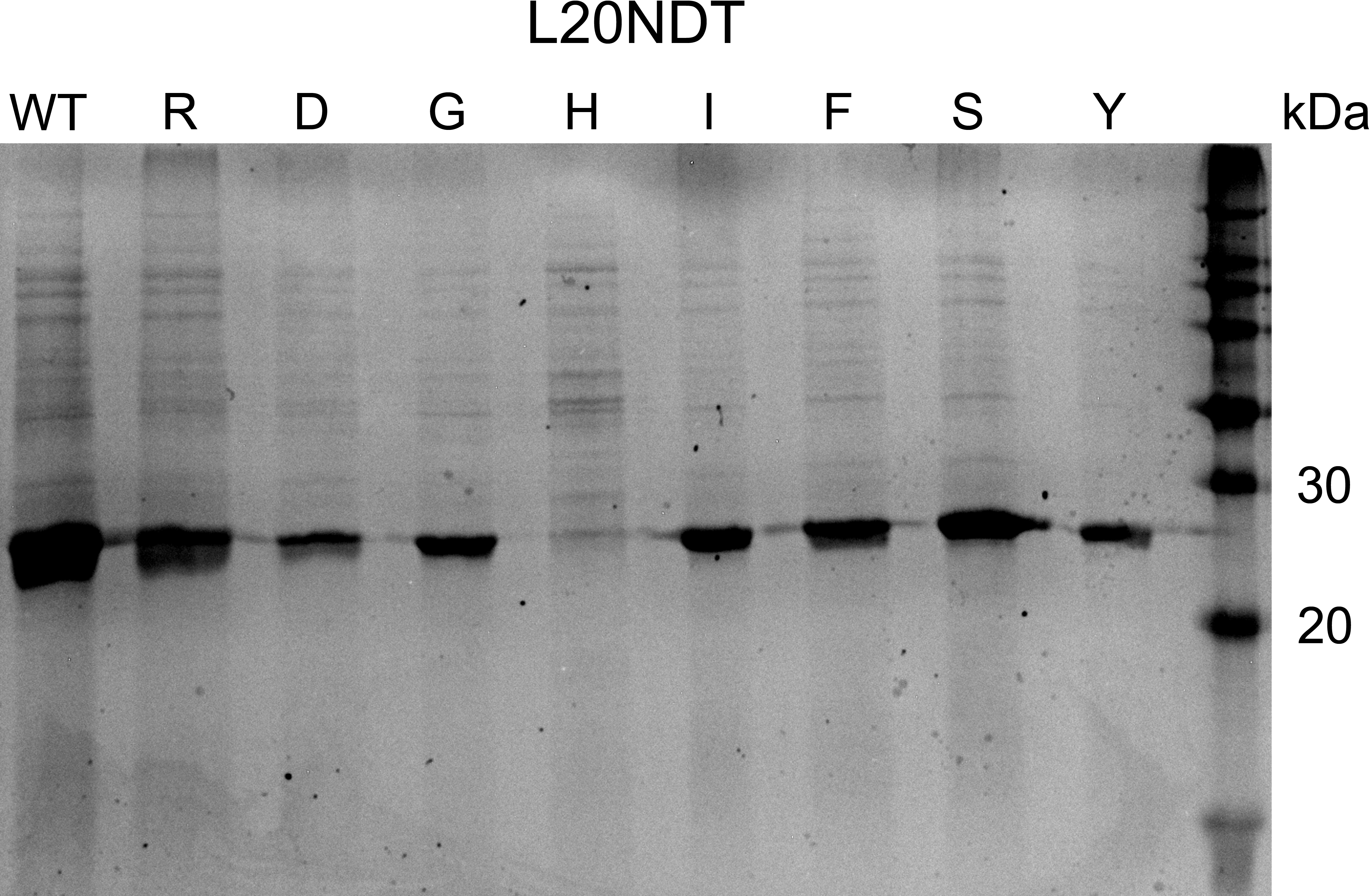

Supplement: S4 Fig — 4–12% Bis-TRIS Gel with colloidal Coomassie stain. 10 μL of a 1:100 dilution of cell-free crude extracts were used, the standard was Roti®-Mark 10–150. WT: DERA wildtype, the mutation is represented in the single-letter code. (TIF) [file pone.0156525.s004.tif]

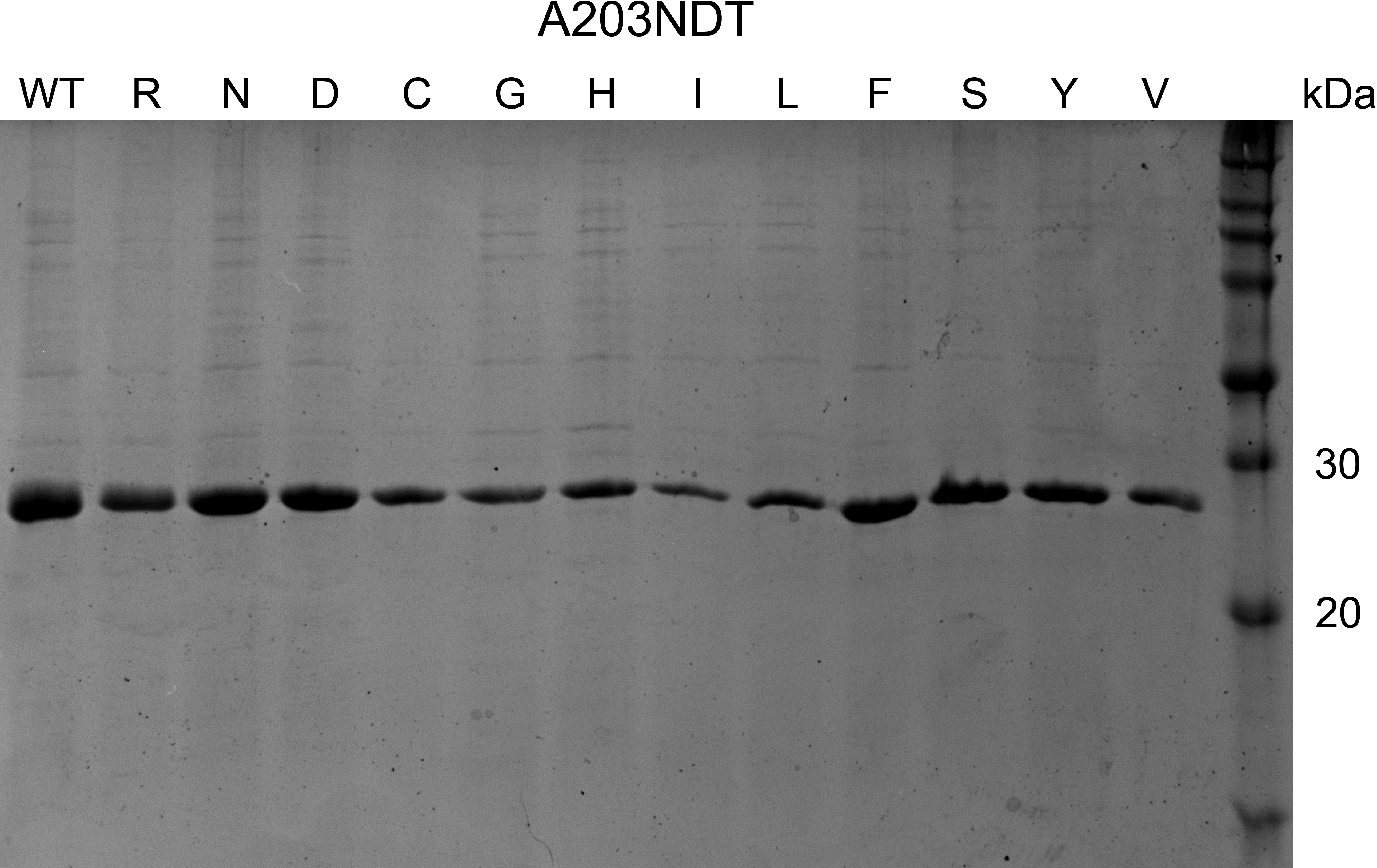

Supplement: S5 Fig — 4–12% Bis-TRIS Gel with colloidal Coomassie stain. 10 μL of a 1:100 dilution of cell-free crude extracts were used, the standard was Roti®-Mark 10–150. WT: DERA wildtype, the mutation is represented in the single-letter code. (TIF) [file pone.0156525.s005.tif]

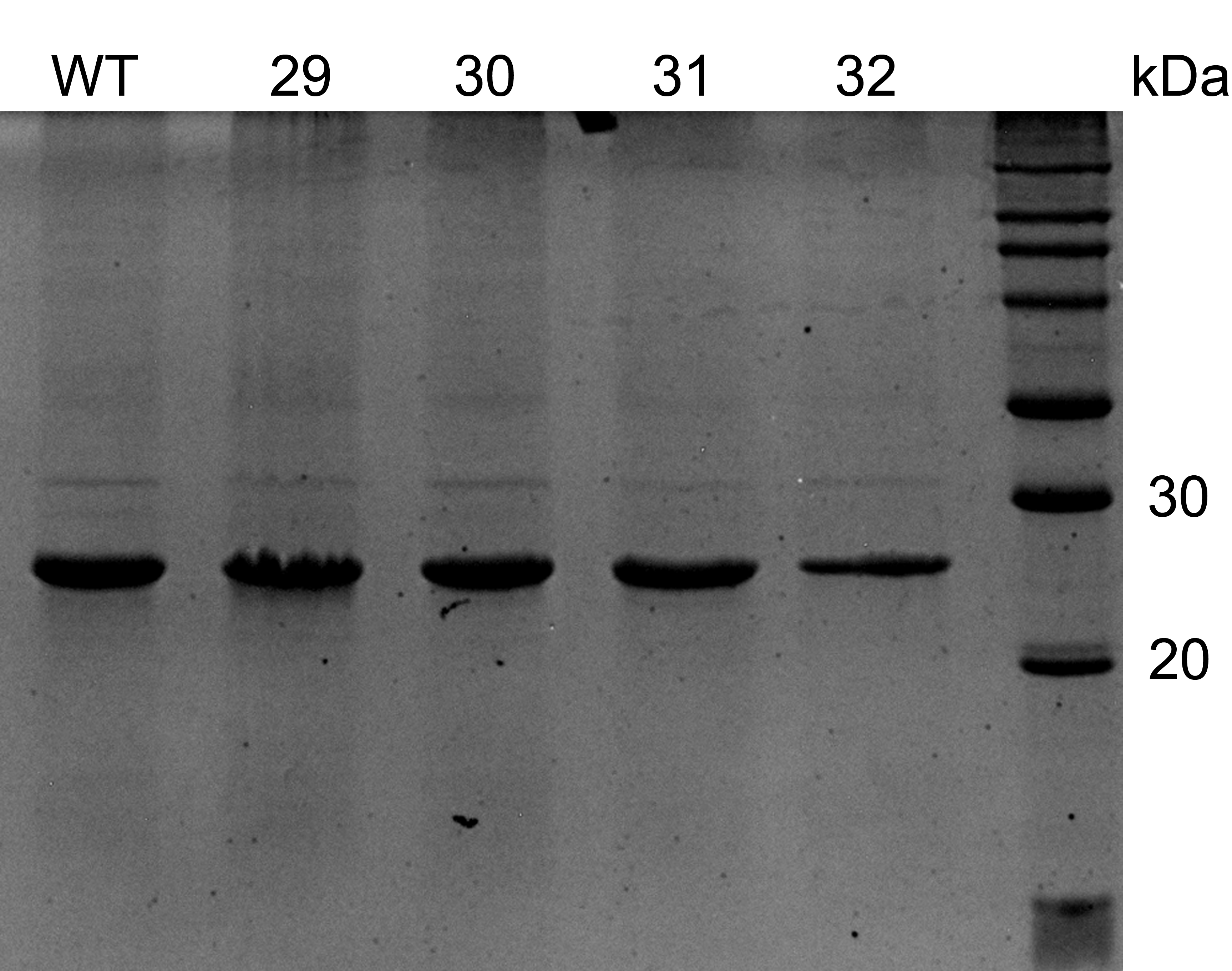

Supplement: S6 Fig — 4–12% Bis-TRIS Gel with colloidal Coomassie stain. 10 μL of a 1:100 dilution of cell-free crude extracts were used, the standard was Roti®-Mark 10–150. WT: DERA wildtype, 29: DERA A203G/T18S, 30: DERA A203G/T18A, 31: DERA A203G/T18I, 32: DERA A203G/T18V. (TIF) [file pone.0156525.s006.tif]

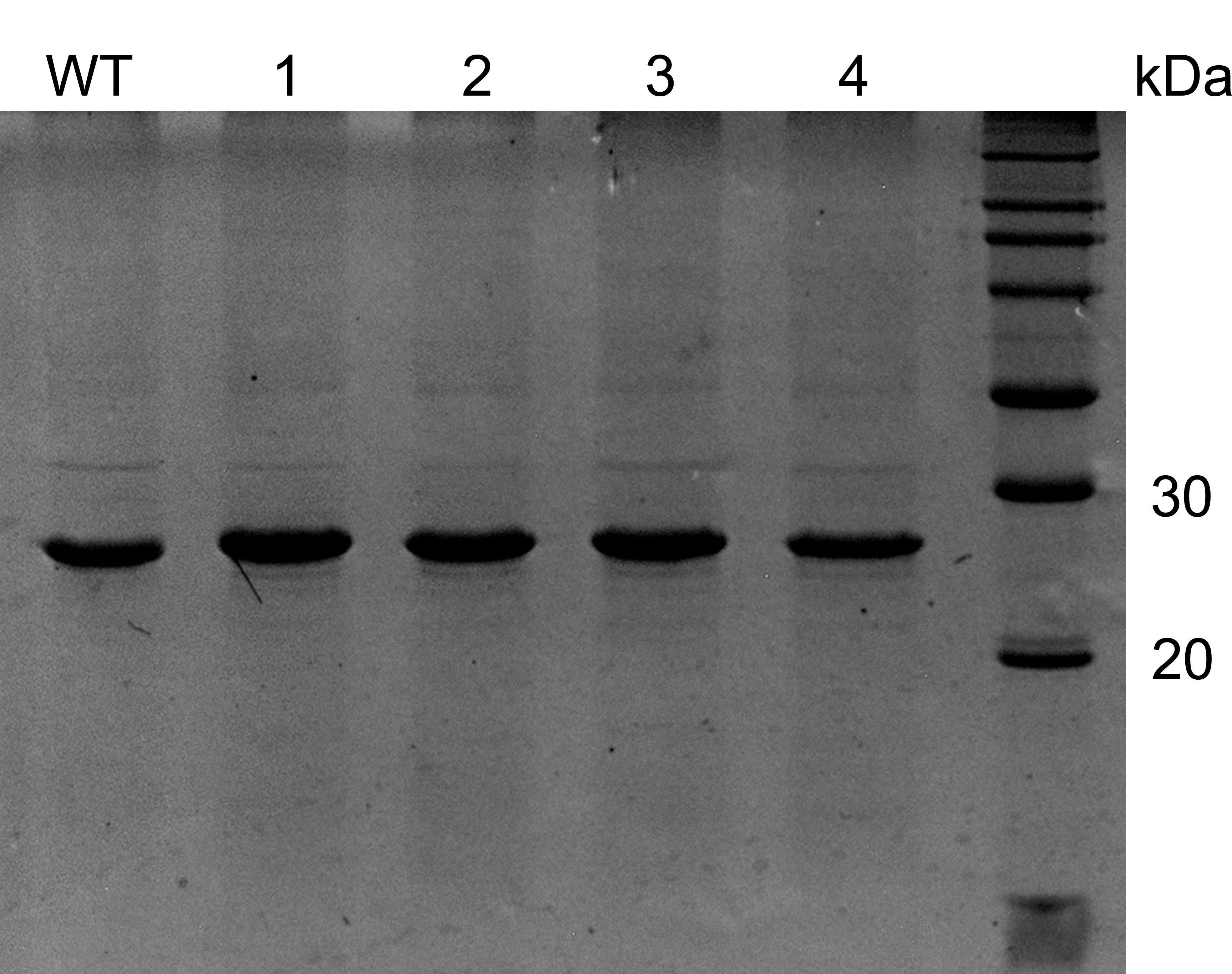

Supplement: S7 Fig — 4–12% Bis-TRIS Gel with colloidal Coomassie stain. 10 μL of a 1:100 dilution of cell-free crude extracts were used, the standard was Roti®-Mark 10–150. WT: DERA wildtype, 1: DERA ΔG204, 2: ΔG204/ΔG205, 3: A203G/ΔG204, 4: A203G/ΔG204/ΔG205. (TIF) [file pone.0156525.s007.tif]

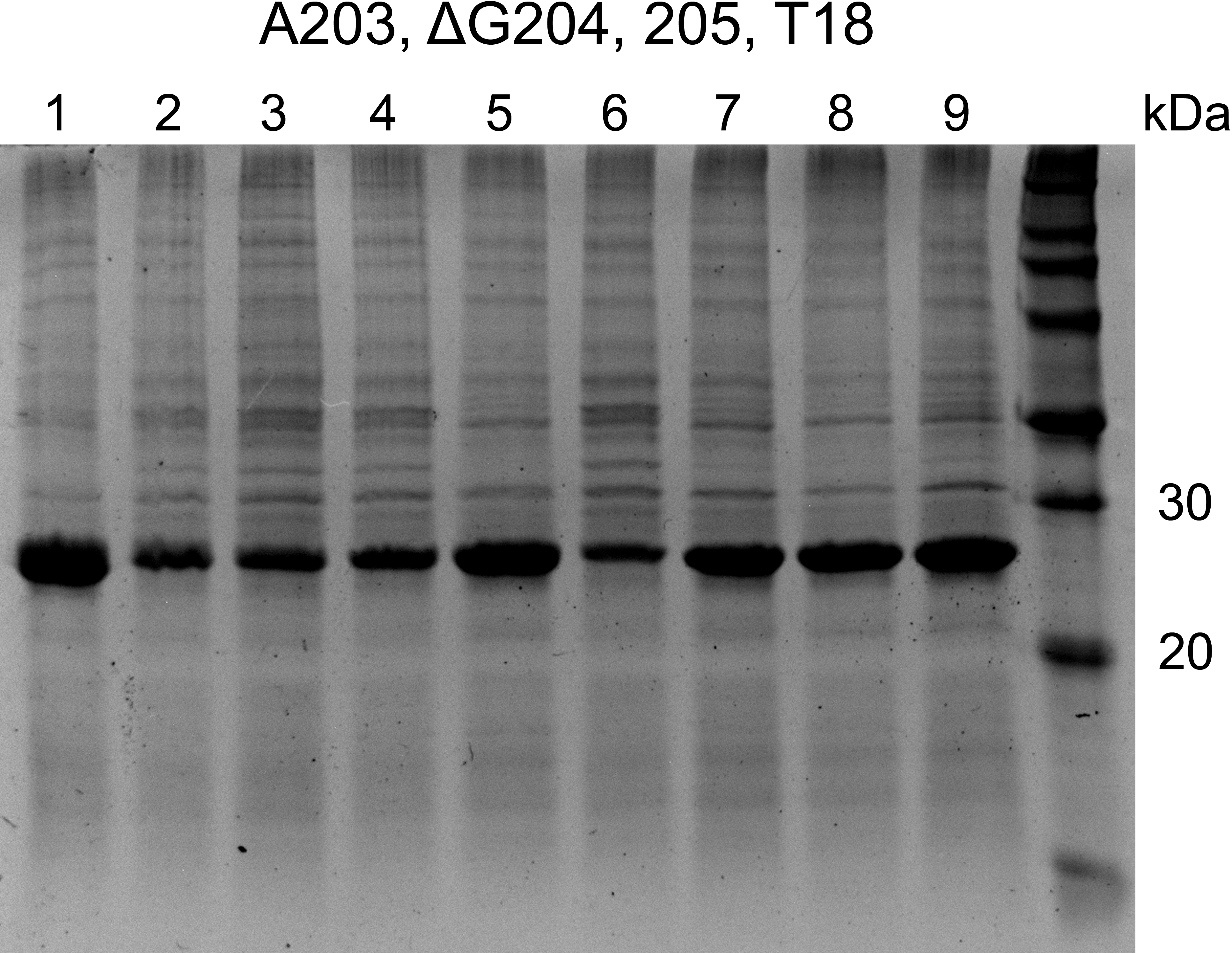

Supplement: S8 Fig — 4–12% Bis-TRIS Gel with colloidal Coomassie stain. 10 μL of a 1:100 dilution of cell-free crude extracts were used, the standard was Roti®-Mark 10–150. 1: DERA wildtype, 2: A203G/ΔG204/T18I, 3: A203G/ΔG204/T18V, 4: A203G/ΔG204/T18A, 5: A203G/ΔG204/T18S, 6: A203G/ΔG204/ΔG205/T18I, 7: A203G/ΔG204/ΔG205/T18V, 8: A203G/ΔG204/ΔG205/T18A, 9: A203G/ΔG204/ΔG205/T18S. (TIF) [file pone.0156525.s008.tif]
