# Supplementary material for: Redesigning Aldolase Stereoselectivity by Homologous Grafting
Source: PLoS One. 2016 Jun 21;11(6):e0156525. doi: 10.1371/journal.pone.0156525 (PMC4915726; doi:10.1371/journal.pone.0156525)
Supplement: S1 Table — [Phos] indicates a phosphorylation at the 5’ position. (PDF) [file pone.0156525.s009.pdf]

S1 Table. Primers used for creation of *KDPG, KDPGal, and DERA* enzyme variants. [Phos] indicates a phosphorylation at the 5' position.

#	name	5'-3'-sequence
1	dgoaEC_chimI_fw	[Phos]CCGGTTATCCGCGGTATTACGCCCGA
2	dgoaEC_chimI_rv	[Phos]TACAACCGGGAGTTTAGTTTGCCA
3	dgoaEC_V154T_fw	[Phos]GCCACCGGCGGCGTGACGCC
4	dgoaEC_V154T_rv	[Phos]AAAGACTGCGATGTCCGATG
5	dgoaEC_NdeI_fw	AACATATGCAGTGGCAAACTAAACTCCCG
6	dgoaEC_XhoI_rv	AAACTCGAGTTGCACTGCCTCTCGATACGC
7	edaEC_chimI_fw	GTGGTAAAAAAACTGGAACACGC
8	edaEC_chimI_fw_t	CGCTGATCGCCATTTTGGTGGTAAAAAAACTGGAACACGC
9	edaEC_chimI_rv	GGCCGGTGGTCAGGATTGAT
10	edaEC_chimI_rv_t	CAAAATGGCGATCAGCGGGCCGGTGGTCAGGATTGAT
11	edaEC_NdeI_fw	AAACATATGAAAAACTGGAAAACAAGT
12	edaEC_T161V_fw	TTTCTCCGGCTAACTACCGTG
13	edaEC_T161V_fw_t	TCTGCCCGGTGGGTGGTATTTCTCCGGCTAACTACCGTG
14	edaEC_T161V_rv	AACGGACCTGGGAGAACGGAC
15	edaEC_T161V_rv_t	TACCACCCACCGGGCAGAAACGGACCTGGGAGAACGGAC
16	edaEC_XhoI_rv	AAACTCGAGCAGCTTAAGCG
17	deoC$_{EC}$_T18R_fwd	[Phos]AGAACCCTGAATGACGACGACACCG
18	deoC$_{EC}$_T18N_fwd	[Phos]AACACCCTGAATGACGACGACACCG
19	deoC$_{EC}$_T18D_fwd	[Phos]GATACCCTGAATGACGACGACACCGA
20	deoC$_{EC}$_T18C_fwd	[Phos]TGCACCCTGAATGACGACGACACC
21	deoC$_{EC}$_T18G_fwd	[Phos]GGCACCCTGAATGACGACGACACC
22	deoC$_{EC}$_T18H_fwd	[Phos]CATACCCTGAATGACGACGACACCG
23	deoC$_{EC}$_T18L_fwd	[Phos]TTAACCCTGAATGACGACGACACCG
24	deoC$_{EC}$_T18F_fwd	[Phos]TTTACCCTGAATGACGACGACACCGA
25	deoC$_{EC}$_T18Y_fwd	[Phos]TATACCCTGAATGACGACGACACCGAC
26	deoC$_{EC}$_T18_rev	[Phos]CAGGTCCATCAATTTCAGTGCACGC
27	deoC$_{EC}$_T18I_fwd	GATGGACCTGATTACCCTGAATG
28	deoC$_{EC}$_T18I_rev	CATTCAGGGTAATCAGGTCCATC
29	deoC$_{EC}$-T18S_fwd	GATGGACCTGTCAACCCTGAATG
30	deoC$_{EC}$-T18S_rev	CATTCAGGGTTGACAGGTCCATC
31	deoC$_{EC}$-T18V_fwd	GATGGACCTGGTAACCCTGAATG
32	deoC$_{EC}$-T18V_rev	CATTCAGGGTTACCAGGTCCATC
33	deoC$_{EC}$-T18A_fwd	GATGGACCTGGCAACCCTGAATG
34	deoC$_{EC}$-T18A_rev	CATTCAGGGTTGCCAGGTCCATC
35	deoC$_{EC}$_L20R_fwd	[Phos]AGAAATGACGACGACACCGACGAG
36	deoC$_{EC}$_L20D_fwd	[Phos]GATAATGACGACGACACCGACGAGAAA
37	deoC$_{EC}$_L20G_fwd	[Phos]GGCAATGACGACGACACCGACG
38	deoC$_{EC}$_L20H_fwd	[Phos]CATAATGACGACGACACCGACGAGA
39	deoC$_{EC}$_L20I_fwd	[Phos]ATTAATGACGACGACACCGACGAGAAA
40	deoC$_{EC}$_L20F_fwd	[Phos]TTTAATGACGACGACACCGACGAGAAA
41	deoC$_{EC}$_L20S_fwd	[Phos]TCGAATGACGACGACACCGACGA
42	deoC$_{EC}$_L20Y_fwd	[Phos]TATAATGACGACGACACCGACGAGAAA
43	deoC$_{EC}$_L20V_fwd	[Phos]GTGAATGACGACGACACCGACGAG
44	deoC$_{EC}$_L20_rev	[Phos]GGTGGTCAGGTCCATCAATTTCAGTGCA
45	deoC$_{EC}$_A203_fwd	[Phos]GGCGTGCGTACTGCGGAAGAT

#	name	5’-3’-sequence
46	deoC$_{EC}$_A203NDT_rev	[Phos]GCCAHNCGGTTTGAAACCAACGGTTTTTTC
47	deoC$_{EC}$_A203R_rev	[Phos]GCCGCGCGGTTTGAAACCAAC
48	deoC$_{EC}$_A203C_rev	[Phos]GCCGCACGGTTTGAAACCAACGG
49	deoC$_{EC}$_A203S_rev	[Phos]GCCCGACGGTTTGAAACCAACGGT
50	deoC$_{EC}$_A203D_rev	[Phos]GCCATCCGGTTTGAAACCAACGGTTTTT
51	deoC$_{EC}$_ΔG204_fwd	[Phos]GGCGTGCGTACTGCGGAAGAT
52	deoC$_{EC}$_ΔG204,ΔG205_fwd	[Phos]GTGCGTACTGCGGAAGATGCG
53	deoC$_{EC}$_A203_rev	[Phos]CGCCGGTTTGAAACCAACGGTTTTTTC
54	deoC$_{EC}$_A203G_rev	[Phos]ACCCGGTTTGAAACCAACGGTTTTTTCTAC
55	deoC$_{EC}$_β-sheet1_fwd	[Phos]GCCATTTTGCTGAATGACGACGACACCGACGAG
56	deoC$_{EC}$_β-sheet1_rev	[Phos]GATCAGCAATTTCAGTGCACGCAGGCTGC
57	deoC$_{EC}$_β-sheet7_fwd	[Phos]GCCGTTGGCGGCGTGCGTACTGC
58	deoC$_{EC}$_β-sheet7_rev	[Phos]TTTGACACCAACGGTTTTTTCTACGCCCAT
59	deoC$_{EC}$_EcoRI_rev	CCGGAATTCTTAGTAGCTGCTGGCGCTCTTACCG
60	deoC$_{EC}$_NdeI_fwd	GGGAATTCCATATGACTGATCTGAAAGCAAGCAGCCTGCG
